# miR-23a/b clusters are not essential for the pathogenesis of osteoarthritis in mouse aging and post-traumatic models

**DOI:** 10.3389/fcell.2022.1043259

**Published:** 2023-01-04

**Authors:** Yusuke Fujiwara, Chenyang Ding, Yohei Sanada, Dilimulati Yimiti, Masakazu Ishikawa, Tomoyuki Nakasa, Naosuke Kamei, Kazunori Imaizumi, Martin K. Lotz, Takayuki Akimoto, Shigeru Miyaki, Nobuo Adachi

**Affiliations:** ^1^ Department of Orthopaedic Surgery, Graduate School of Biomedical & Health Sciences, Hiroshima University, Hiroshima, Japan; ^2^ Medical Center for Translational and Clinical Research, Hiroshima University Hospital, Hiroshima, Japan; ^3^ Department of Artificial Joints and Biomaterials, Graduate School of Biomedical and Health Sciences, Hiroshima University, Hiroshima, Japan; ^4^ Department of Biochemistry, Graduate School of Biomedical and Health Sciences, Hiroshima University, Hiroshima, Japan; ^5^ Department of Molecular Medicine, Scripps Research, La Jolla, CA, United States; ^6^ Faculty of Sport Sciences, Waseda University, Tokorozawa, Japan

**Keywords:** osteoarthritis, microRNA, cartilage, genetically modified mice, mouse model, bone, aging

## Abstract

Osteoarthritis (OA), the most prevalent aging-related joint disease, is characterized by insufficient extracellular matrix synthesis and articular cartilage degradation and is caused by various risk factors including aging and traumatic injury. Most microRNAs (miRNAs) have been associated with pathogenesis of osteoarthritis (OA) using *in vitro* models. However, the role of many miRNAs in skeletal development and OA pathogenesis is uncharacterized *in vivo* using genetically modified mice. Here, we focused on miR-23–27–24 clusters. There are two paralogous miR-23–27–24 clusters: miR-23a-27a-24–2 (miR-23a cluster) and miR-23b-27b-24–1 (miR-23b cluster). Each miR-23a/b, miR-24, and miR-27a/b is thought to function coordinately and complementary to each other, and the role of each miR-23a/b, miR-24, and miR-27a/b in OA pathogenesis is still controversial. MiR-23a/b clusters are highly expressed in chondrocytes and the present study examined their role in OA. We analyzed miRNA expression in chondrocytes and investigated cartilage-specific miR-23a/b clusters knockout (Col2a1-Cre; miR-23a/b*flox/flox*: Cart-miR-23clus KO) mice and global miR-23a/b clusters knockout (CAG-Cre; miR-23a/b*flox/flox*: Glob-miR-23clus KO) mice. Knees of Cart- and Glob-miR-23a/b clusters KO mice were evaluated by histological grading systems for knee joint tissues using aging model (12 and/or 18 month-old) and surgically-induced OA model. miR-23a/b clusters were among the most highly expressed miRNAs in chondrocytes. Skeletal development of Cart- and Glob-miR-23clus KO mice was grossly normal although Glob-miR-23clus KO had reduced body weight, adipose tissue and bone density. In the aging model and surgically-induced OA model, Cart- and Glob-miR-23clus KO mice exhibited mild OA-like changes such as proteoglycan loss and cartilage fibrillation. However, the histological scores were not significantly different in terms of the severity of OA in Cart- and Glob-miR-23clus KO mice compared with control mice. Together, miR-23a/b clusters, composed of miR-23a/b, miR-24, miR-27a/b do not significantly contribute to OA pathogenesis.

## 1 Introduction

Osteoarthritis (OA) is the most common musculoskeletal disorder caused by risk factors including aging, traumatic injury, and obesity. However, the pathogenesis of OA has not been characterized completely, so currently, there are limited treatment options available for OA prevention or disease modification. Although OA has been considered a disease of cartilage degradation, a recent study indicated that OA is a systemic disease or a whole joint disease accompanied by changes of joint tissues such as subchondral bone (ScB) sclerosis, meniscus degeneration and synovial inflammation (Loeser et al., 2012; Cicuttini and Wluka, 2014; June et al., 2016).

MicroRNAs (miRNAs) are a class of non-coding RNAs involved in fundamental mammalian homeostasis and various diseases through regulating post-transcriptional regulation (Bartel, 2018). It has been reported that various miRNAs regulate chondrocyte homeostasis and inflammatory signaling cascade which are associated with OA pathogenesis (Endisha et al., 2018). Among them, miR-140, which is highly and specifically expressed in cartilage, regulates skeletal development and cartilage homeostasis, and is involved in OA development (Miyaki et al., 2009; Miyaki et al., 2010; Nakamura et al., 2011). Although several miRNAs, such as miR-140 and miR-455, demonstrate the potential to be OA regulatory factors and attractive therapeutical options (Miyaki et al., 2009; Miyaki et al., 2010; Wang et al., 2017; Huang et al., 2019; Duan et al., 2020; Ito et al., 2021), almost all of the studies are analyses by cultured chondrocytes and knee injection with mimic and antioligo for miRNA so far. However, their effects are transient and limited, especially in skeletal development and aging models. Thus, the approaches using genetically modified mice with gain- and loss-of-function model have an advantage in understanding the functions of miRNAs on skeletal and OA development. miR-23a/b clusters, composed of miR-23a–27a–24–2 (miR-23a cluster) and miR-23b–27b–24–1 (miR-23b cluster) are two paralogous miRNA that are located on chromosomes 8 and 13, respectively, in the mouse genome and on chromosomes 19 and 9, respectively, in the human genome. The miRNAs in each of the two clusters of interest are 100% homologous between human and mouse. Each individual miRNA (miR-23a, miR-23b, miR-24, miR-27a, and miR-27b) is finally processed from each cluster for the display of their function as matured miRNAs. Previous reports indicate that among miR-23a/b clusters, miR-23a, miR-24, and miR-27b have a role in maintaining articular cartilage integrity (Akhtar et al., 2010; Philipot et al., 2014; Hu et al., 2017; Zhou et al., 2017; Xu et al., 2018; Lv et al., 2020; Xu et al., 2021). MiR-27, similar to miR-140, is downregulated in patients with OA (Akhtar et al., 2010; Zhou et al., 2017; Xu et al., 2018). On the other hand, miR-23a and miR-23b have been reported to contribute to OA progression (Kang et al., 2016; Guo et al., 2018; Zhao et al., 2019). Non-etheless, these reports have come from cultured chondrocytes with single miRNA mimic or inhibitor such as miR-23a. Each miR-23a/b, miR-24, and miR-27a/b is thought to function coordinately and complementary to each other because they are existing as clusters. Thus, the role of each miRNA that composes miR-23a/b clusters is still controversial in OA pathogenesis. Furthermore, recently, not only cellular miRNAs but also exosomal miRNAs in extracellular vesicles (EVs) such as exosomes have attracted attention for their relation to the pathogenesis, as diagnostic markers, and in the treatment of OA (Miyaki and Lotz, 2018; Mihanfar et al., 2020). The exosomal miR-23a, miR-23b, miR-24, and miR-27b in plasma have been shown to have the potential for development of diagnosis, pathogenesis, and treatments of various diseases including arthritis (Castro-Villegas et al., 2015; Liu et al., 2019; Garavelli et al., 2020; Castanheira et al., 2021). Thus, the role of miR-23a/b clusters in OA pathogenesis firstly requires elucidation by a loss-of-function approach of miR-23a/b clusters using mouse model.

The purpose of the present study is to determine the role of miR-23a/b clusters in OA pathogenesis using cartilage-specific- and global-miR-23a/b clusters deficient mice in two different mouse models of OA, an aging model, and a surgical model.

## 2 Materials and methods

### 2.1 Human articular cartilage tissues

Human tissue collection was approved by Human Subjects Committee of Scripps Research or Hiroshima University Hospital. Human articular cartilages from the intact knee joints were procured by tissue banks from 6 donors (mean ± SD age 31.0 ± 15.5 years: female 5, male 1). Cartilages were obtained from 14 patients with OA (mean ± SD age 72.3 ± 7.5 years: female 11, male 3) who were undergoing total knee arthroplasty. All samples were examined by Safranin O staining and graded according to a modified Mankin scale, with a score of < 2 points for normal and a score of > 5 for severe OA. Human chondrocytes were isolated and cultured from articular cartilage as described previously (Maier et al., 1993). The effect size in the analysis (Normal *n* = 6 vs. OA *n* = 14) was 1.45 with the statistical power of .8 (G power 3.1).

### 2.2 Nanostring nCounter microRNA analysis

Nanostring nCounter microRNA analysis was performed using RNA from human articular chondrocytes and human articular chondrocytes-derived exosomes as described previously (Furuta et al., 2016). Human articular chondrocytes were cultured with Dulbecco’s modified Eagle’s medium (DMEM)/10% FBS. One day later, the cells were washed with serum-free DMEM and cultured with serum-free DMEM for 48 h. To isolate the exosomes, 2 ml of conditioned medium was collected and centrifuged for 15 min at 2,380G and then further ultracentrifuged for 70 min at 180,000G at 4°C. The exosome were pellets were resuspended in 100 ml of PBS. The exosomes isolated from the same volume of culture mediums and from the same numbers of cells. Small RNAs were purified from exosome of conditioned medium of cultured human articular chondrocytes using the mirVana miRNA Isolation Kit (Thermo Fisher Scientific Life Sciences, Oakwood Village, OH, https://www.thermofisher.com). Purified small RNAs were concentrated using an evaporator. The concentrations and quality of the small RNAs from the same volume of culture mediums for the same numbers of cells were determined by the BioAnalyzer 2,100 (Agilent Technologies, Santa Clara, CA, http://www.agilent.com), and small RNA was used as the input for the nCounter Human miRNA Expression Assay Kit (NanoString Technologies, Seattle, WA, http://www.nanostring.com) according to manufacturer’s instruction.

### 2.3 Animal study

All animal studies were performed according to protocols approved by Institutional Animal Care and Use Committee at Hiroshima University. All mice were housed in temperature-controlled quarters (23°C ± 1°C) with a 12-h light-dark cycle and in groups of two to five per cage (143 mm × 293 mm × H148 mm) and were freely allowed access to food and water.

### 2.4 Generation of cartilage-specific miR-23a/b clusters KO mice and global miR-23a/b clusters KO mice

Cartilage-specific miR-23a/b clusters KO (Cart-miR23clus KO) mice and Global miR-23a/b clusters KO (Glob-miR23clus KO) mice were generated by crossbreeding the previously described *Col2*Cre-driver mice (*Col2*Cre mice)(Ovchinnikov et al., 2000) or *CAG*Cre-driver mice (*CAG*Cre mice)(Matsumura et al., 2004) and miR-23a/b clusters floxed mice on C57BL6/J background (Oikawa et al., 2018; Lee et al., 2019). The miR-23a/b clusters^flox/flox^ mice were used as Control. To confirm the miR-23a/b clusters deficiency, we performed the genotyping PCR following PCR primer sets (Supplementary Table S1) as previous reported (Oikawa et al., 2018; Lee et al., 2019). In aging study, we measured the body weight during aging, and body length was measured at the end points of the experiment. Knee joints were harvested at 12 months and/or 18 months of age to monitor spontaneous age-related OA. Control and Cart-miR-23clus KO mice (*n* = 7, *n* = 7), and Control and Glob-miR-23clus KO mice (*n* = 11, *n* = 13) at 12 months of age, and Control and Cart-miR-23clus KO mice (*n* = 12, *n* = 12) at 18 months of age were assessed by histological scoring systems. Experimental OA was induced in Control and Cart-miR23clus KO mice at 12 weeks of age by performing medial meniscectomy and transection of the medial collateral ligament (MCL) in the right knees (Kamekura et al., 2005). Mice were sacrificed at 8 weeks (Control: *n* = 8, Cart-miR-23clus KO: *n* = 8) and 12 weeks (Control: *n* = 12, Cart-miR-23clus KO: *n* = 11) after surgery, and the right knee joints were collected for histological analysis. In the present study, a total of 4 wild type (C57BL6/J), 73 Control, 60 Cart-miR23clus KO and 21 Glob-miR23clus KO mice were sacrificed for histopathological assessment and *in vitro* experiments. All experiments were performed using male mice. Sample sizes were chosen based on prior literature using similar methods (Chang et al., 2019). The previous report demonstrated moderate effect size (> 1.7) in aging model (Control mice *n* = 8 vs. KO mice *n* = 8) and moderate effect size (> 1.5) in surgically-induced OA model (Control mice *n* = 9 vs. KO mice *n* = 9). We determined the sample size appropriate to maintain the effect size of 1.5 with statistical power of .8 (G power 3.1) and performed a minimum of *n* = 7. However, we were determined to perform *n* = 12 considering issues such as life span of genetically modified mice and loss of mice due to various causes in aging model of 18 months of age.

### 2.5 Histopathological assessments

Whole-mount Alcian blue (Sigma-Aldrich, United States) and Alizarin red S (Sigma-Aldrich, United States) staining of skeletons were performed on Control, Cart-miR23clus KO and Glob-miR23clus KO mice at postnatal day 0. All knee joints were embedded intact in paraffin after fixation in 4% paraformaldehyde phosphate buffer solution (PBS) and decalcification in K-CX or EDT-X (FALMA, Japan). Knee joints were sectioned (4.5 μm) in the coronal plane anterior to posterior through the central weight-bearing region of the femorotibial joint. The sections were stained with Safranin O (MUTO PURE CHEMICALS, Tokyo, Japan) and Fast Green (Sigma-Aldrich, United States) and three different sections per joint were analyzed microscopically. Three different researchers were blinded while performing all manual scorings. Damage to the articular cartilage (maximum of 24 points per knee joint section; 6 points for each quadrant of the tibial/femoral cartilage) was evaluated using the OARSI scoring system (Glasson et al., 2010). Subchondral bone changes, meniscus degradation and the severity of synovitis were evaluated using the right knee joints according to previously described histopathological scoring systems (Krenn et al., 2002; Kwok et al., 2016; Nagira et al., 2020).

### 2.6 Immunohistochemical analysis

Slides were pretreated with antigen-retrieval reagent (Immunoactive; Matsunami Glass Ind, Osaka, Japan) at 60°C for 16 h, followed by blocking serum for 30 min. Then, sections were immunostained with anti-P16^INK4a^ antibody (abcam, ab54210, 0.1 μg/ml), anti-ADAMTS5 antibody (GeneTex, GTX100332, 10 μg/ml) and anti-MMP13 antibody (ThermoFisher Scientific, MA5-14328, 20 μg/ml) diluted in Can Get Signal immunostaining solution (TOYOBO, Tokyo, Japan) using Vectastain ABC-AP alkaline phosphatase kit and AP substrate kit (Vector Laboratories, Burlingame, CA, United States) according to the manufacturers’ instructions. For type II and type X collagen staining, slides were pretreated with antigen retrieval reagent (Proteinase K, Dako, CA, United States) at room temperature for 10 min and blocking serum for 30 min. Then, sections were immunostained with anti-type II collagen antibody (DSHB, CIIC1, 6 μg/ml) and anti-type X collagen antibody (DSHB, X-AC9, 5 μg/ml) diluted in PBS using Vectastain Elite ABC-HRP kit and DAB substrate kit. All stainings and evaluations were performed using *n* = 5 each group.

### 2.7 Mouse chondrocytes isolation and culture

Articular cartilage from the femoral heads of mice at 3 weeks of age was taken, and chondrocytes were isolated by digestion with 3.5 mg/ml collagenase Type 2 (Worthington, Lakewood, NJ, United States) in Dulbecco’s modified Eagle’s medium (DMEM) (FUJIFILM Wako, Osaka, Japan) for 1.5 h at 37°C. Isolated chondrocytes were cultured in DMEM with 10% fetal bovine serum. Experiments were carried out at passage 1. Chondrocytes were treated with or without IL-1β (1 ng/ml; Pepro-Tech, Rocky Hill, NJ, United States) for 24 h.

### 2.8 Quantitative real-time PCR

Total RNA was extracted from various tissues and cultured chondrocytes using Isogen reagent (Nippon gene, Tokyo, Japan) and RNA purification kit (Direct-zol RNA microprep, Zymo Research, California, United States). Small RNA from serum was extracted using Maxwell RSC miRNA Plasma and Serum kit (Promega, Wisconsin, United States). Complementary DNA (cDNA) was synthesized with a Reverse Transcription system (iScript supermix, BioRad, California, United States) according to the manufacturer’s protocol. Quantitative polymerase chain reaction (PCR) was performed with the TaqMan Gene Expression Assay probes (Supplementary Table S2) (Thermo Fisher Scientific, Massachusetts, United States). *Gapdh* and U6 snRNA were used as the internal controls to normalize the sample differences. Relative expression was calculated using the ΔΔCt values, and results were expressed as 2^−ΔΔCt^.

### 2.9 Glycosaminoglycan release assay

Femoral head cartilages (femoral cap: Control *n* = 8, Cart-miR-23clus KO *n* = 7) were harvested from 3-week-old Control and Cart-miR-23a/b clusters KO mice and weighed. The amount of the released glycosaminoglycan into medium was measured using the Blyscan Glycosaminoglycan assay kit (Biocolor, United Kingdom) as previously described (Ishitobi et al., 2018). Size variance between femoral caps was normalized by their weight.

### 2.10 Immunoblotting analysis

Protein was extracted from cultured chondrocytes using M-PER™ protein extraction reagent (Thermo Fisher Scientific) with protease inhibitor cocktail set I (FUJIFILM Wako) and phosphatase inhibitor cocktail I (abcam). Total protein (20 μg–30 μg) was separated by SDS/PAGE (10%), and electrically transferred onto PVDF membranes (BioRad). After blocking with 5% skim milk in TBST, the membranes were incubated with primary antibodies and then incubated with secondary antibodies. The signal was detected with chemiluminescent of enhanced immunostar SD (FUJIFILM Wako). The quantifications of immunoblotting were performed using ImageJ (version 1.53o). Antibodies used in immunoblotting are listed in Supplementary Table S3. All immunoblottings were performed using *n* = 5 each group.

### 2.11 DEXA analysis

The skin and muscles were removed from the hind limbs which were fixed in 4% paraformaldehyde phosphate buffer solution (PBS) for 48 h at 4°C. Bone mineral density (BMD) in femur bone (*n* = 7 each group: Control and Glob-miR-23clus KO mice at 12 months of age) was measured using dual-energy X-ray absorptiometry (DEXA) densitometry (Aloka DCS-600EX, Aloka Co., Tokyo, Japan). Bone density measurements of left femur were taken from sixteen consecutive images with a scan pitch of 2 mm by DEXA scan from the distal femur.

### 2.12 TUNEL staining

TUNEL staining was completed using an *in-situ* detection kit for programmed cell death detection (MEBSTAIN apoptosis TUNEL Kit direct: MBL, United States) according to the manufacturer’s instructions. Nuclei were stained by 4′,6-diamidino-2-phenylindole (DAPI). TUNEL staining were performed using *n* = 5 each group.

### 2.13 Multiomics analysis

Articular chondrocytes from the femoral heads of mice at 3 weeks of age were isolated by digestion with 3.5 mg/ml collagenase Type 2 (Worthington, Lakewood, NJ, United States) in DMEM (FUJIFILM Wako, Osaka, Japan) for 1.5 h at 37°C. Isolated chondrocytes were cultured in DMEM with 10% fetal bovine serum. Primary chondrocytes were harvested from dishes after reaching confluency and seeded in 6-well plates at 5 × 10^5^ cells per well. Total RNA and protein of Control and, Cart-miR-23a/b KO chondrocytes were extracted and underwent Multiomics (RNA-sequencing and DIA proteome) analysis at Kazusa Genome Technologies (Kisarazu, Japan). Quantitative analysis of proteomes was performed by the data-independent acquisition (DIA) proteome analysis using Q-Exactive^™^ HF-X (Thermo Fisher Scientific) as described previously (Kawashima et al., 2019). The mass spectrometry proteomics data that can be accessed have been deposited to the ProteomeXchange Consortium *via* the PRIDE partner repository with the dataset identifier, PXD031868. RNA-seq libraries were prepared using QuantSeq 3′mRNA-Seq Library Prep Kit for Illumina (FWD) (015.384, LEXOGEN). RNA-Seq was carried out using single-end 75 base read sequencing using an Illumina NextSeq500 sequencer. After checking the quality of the reads, it was determined that filtering of low-quality reads was not necessary. The reads were mapped to the mouse reference genome (10 mm) and the expression of the identified genes was normalized by calculation of TPM. The RNA-seq data have been deposited in NCBI’s Gene Expression Omnibus and are accessible through GEO Series accession number, GSE197363 (https://www.ncbi.nlm.nih.gov/geo/query/acc.cgi?acc=GSE197363). Differentially expressed genes were extracted and subsequently imported into gene ontology enrichment analysis with Metascape (https://metascape.org) (Zhou et al., 2019).

### 2.14 Statistical analysis

Actual measurement values of all data are presented as mean ± standard error of mean (SEM) or standard deviation (SD). For comparison between two groups, Welch’s *t*-test or Mann-Whitney *U* test was applied. For multiple comparison, *p*-values were corrected with Holm-Sidak method (Graph Pad Prism 9.0). Scoring data in aging OA model and surgically-induced OA model was analyzed for differences between mouse type and time-points using nonparametric Kruskal-Wallis test, then the *post-hoc* Dunn’s test or Mann-Whitney *U* test to evaluate individual comparisons. Body weight and bone density were analyzed with two-way ANOVA, then *post hoc* Sidak’s test to evaluate individual comparisons. Differences were considered statistically significant at *p* < .05. Sample sizes were chosen based on prior literature using similar methods, reporting moderate effect size, and our previous experiments.

## 3 Results

### 3.1 Expression patterns of miR-23a/b clusters in articular cartilage

**FIGURE 1:**
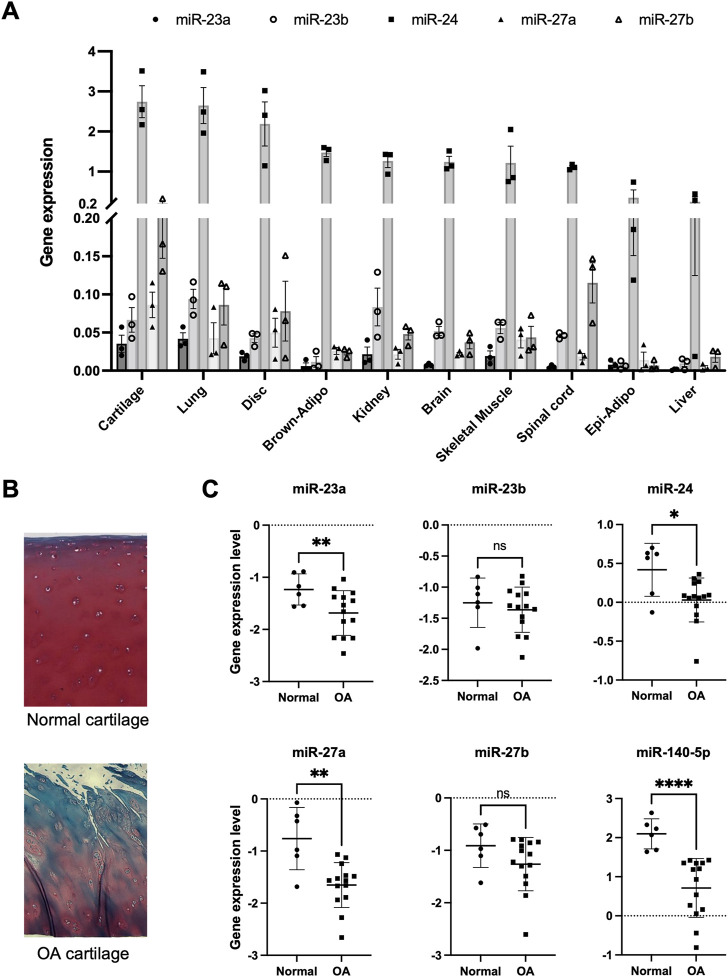
Expression pattern of miR-23a/b clusters in various tissues and osteoarthritis cartilage. **(A)** miR-23a/b clusters were expressed in various tissues (Cartilage: articular cartilage, Lung, Disc: intervertebral disc, Brown-Adipo: brown adipose tissue, Kidney, Brain, Skeletal muscle, Spinal cord, Epi-Adipo: epididymal adipose tissue, and Liver: *n* = 3 each tissue) of C57BL6/J mice at 4 weeks of age by real-time PCR analysis. **(B)** Normal human cartilage and OA cartilage for Isolation of Articular chondrocytes. Representative histological image with Safranin-O staining. **(C)** The expression of miR-23a/b clusters and miR-140 in articular chondrocytes from human normal cartilage (*n* = 6) and OA cartilage (*n* = 14). All data are represented as mean ± SEM. Comparison of mean values was performed by Welch’s *t*-test; *p < .05, **p < .01, ****p < .0001 *versus* normal chondrocytes. n. s: non-significant difference.

To characterize the expression profile of miRNAs in normal human articular chondrocytes, we first performed miRNA expression analysis using Nanostring nCounter (Supplementary Table S4). In this analysis, we focused on miR-23a/b clusters which were expressed at higher levels than miR-140 in chondrocytes. The expression of miR-23a/b clusters was observed in various tissues of C57BL/6J mice at 4 weeks of age, wherein they were expressed more abundantly in the lung and articular cartilage (Figure 1A). Among miR-23a/b clusters, especially miR-24 was highly expressed in various tissues including articular cartilage. These results demonstrated that miR-23a/b clusters were expressed in a broad range of tissues but not in cartilage-specific expression pattern. However, the expression levels of miR-23a/b clusters were especially higher in cartilage including intervertebral disc and lung than other tissues. Furthermore, we examined the expression of miR-23a/b clusters in articular chondrocytes from normal and OA cartilage (Figure 1B). The expression of miR-23a, miR-24 and miR-27a was significantly decreased in OA chondrocytes compared with normal chondrocytes consistent with prior findings about miR-140 (Figure 1C). Thus, previous inconsistent reports (Akhtar et al., 2010; Philipot et al., 2014; Hu et al., 2017; Zhou et al., 2017; Xu et al., 2018; Lv et al., 2020; Xu et al., 2021) and our results prompted us to examine the role of miR-23a/b clusters in cartilage development and OA pathogenesis using miR-23a/b clusters-deficient mice. We expected that 23a/b clusters-deficient mice would exhibit abnormal skeletal development and altered onset or severity of OA.

### 3.2 Cartilage-specific miR-23a/b clusters loss-of-function model

**FIGURE 2:**
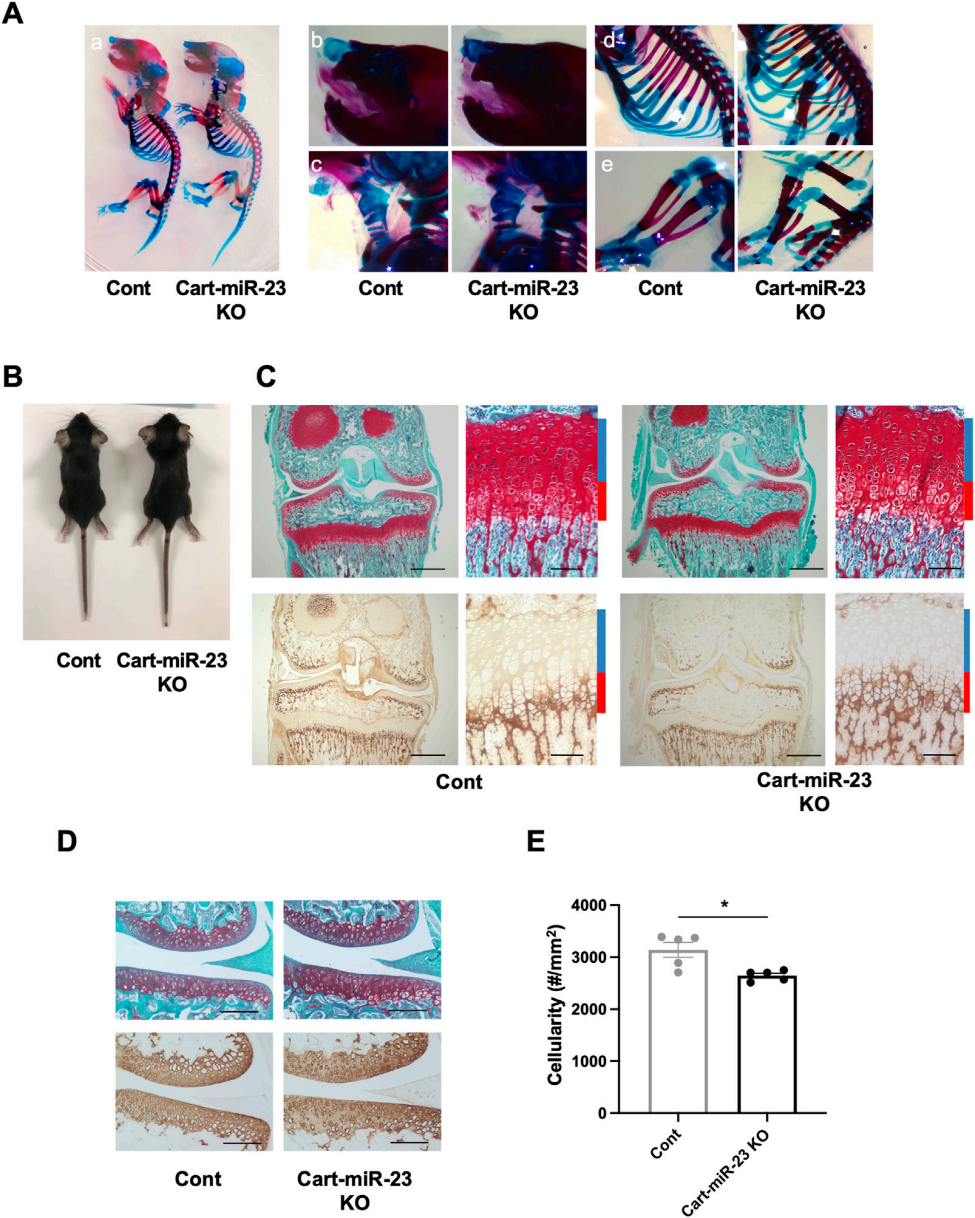
Skeletal development in Cart-miR-23clus KO mice. **(A)** Skeletal preparation with Alcian blue/Alizarin red staining of Control and Cart-miR-23clus KO mice in postnatal day 0. **(A)** Whole mount, **(B)** nasal capsule, **(C)** trachea, **(D)** rib cage, **(E)** hind limb. Cart-miR-23clus KO mice exhibited no overt abnormality in skeletal development. **(B)** Appearance of Control and Cart-miR-23clus KO mice at 3 weeks of age. Growth retardation was not observed in Cart-miR-23clus KO mice. **(C)** Safranin O/Fast Green staining and immunohistochemistry (Type X Collagen) in articular cartilage of knee joints and in the tibial growth plate in Control and Cart-miR-23clus KO mice at 3 weeks of age. Scale bars: 500 μm and 100 µm. **(D)** Safranin O/Fast Green staining and immunohistochemistry (Type II Collagen) in articular cartilage of knee joints in Control and Cart-miR-23clus KO mice at 3 weeks of age. Scale bars: 200 µm. **(E)** Cellularity in articular cartilage of Control and Cart-miR-23clus KO mice (*n* = 5 per group). The data represented as mean ± SEM. Comparison of mean values was performed using Welch’s *t*-test; *p < .05.

The expression pattern of miR-23a/b clusters was ubiquitous. To examine the role of miR-23a/b clusters in cartilage, we performed experiments through a cartilage-specific loss-of-function approach using Cart-miR-23clus KO mice. Cart-miR-23clus KO mice were generated by crossing miR-23a/b clusters floxed mice (Lee et al., 2019) with *Col2*-Cre Tg mice (Ovchinnikov et al., 2000). The expression levels of miR-23a cluster (miR-23a–27a–24–2) and miR-23b cluster (miR-23b–27b–24–1) were decreased specifically in cartilage and chondrocytes of Cart-miR-23clus KO mice compared with Control mice (Supplementary Figures S1A, B). However, Cart-miR-23clus KO mice postnatally showed no difference in skeletal development (Figures 2A–E, B). The articular cartilage and growth plate staining with Safranin O, type X Collagen, and type II Collagen, and the structure and shape of the knee joints and their menisci were normal in Cart-miR-23clus KO mice at 3 weeks of age (Figures 2C, D). Although the cellularity of articular cartilage was significantly decreased in Cart-miR-23clus KO mice compared with Control mice (Figures 2D, E), Cart-miR-23clus KO mice exhibited almost normal skeletal development and maturation.

**FIGURE 3:**
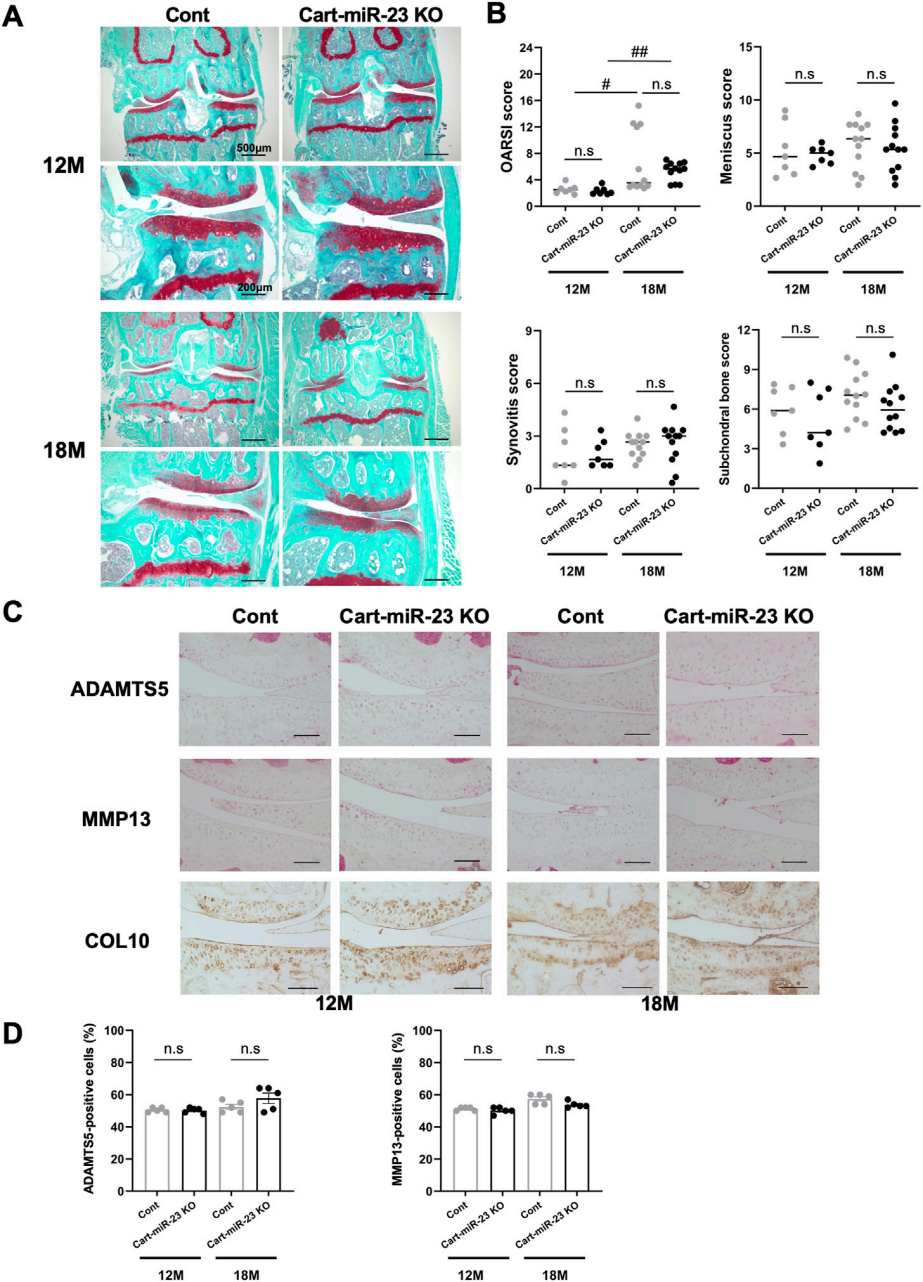
Histopathological evaluation of Knee joint tissues in Cart-miR-23clus KO mice with aging. **(A)** Safranin O staining of the knee joints of Control and Cart-miR-23clus KO mice at 12 and 18 months of age. Scale bars: 500 µm and 200 µm. **(B)** The right knee joints of Control and Cart-miR-23clus KO mice at 12 months of age (Control: *n* = 7, Cart-miR-23clus KO: *n* = 7) and 18 months of age (Control: *n* = 12, Cart-miR-23clus KO: *n* = 12) were assessed by OARSI scoring, meniscus scoring, synovitis scoring and ScB scoring system. The data was represented as median. Scoring data was analyzed by Kruskal–Wallis test, the Dunn’s multiple comparison test was applied; ^#^ *p* < .05, ^##^ *p* < .01. **(C,D)** Knee joints from Control and Cart-miR-23clus KO mice were assessed by immunohistochemistry using anti-ADAMTS5, anti-MMP13, and anti-Type X Collagen antibodies (*n* = 5 per group). Scale bars: 100 µm. The data represented as mean ± SEM. Comparison of mean values was performed using Welch’s *t*-test with Holm-Sidak correction for multiple comparison. n. s: non-significant difference.

To determine whether miR-23a/b clusters have role in the pathogenesis of OA, we examined the knee joints of Cart-miR-23clus KO mice in two different mouse models of OA, an aging model as primary OA, and a surgical model as posttraumatic OA. First, in aged mice, the bodyweight and the body length of Cart-miR-23clus KO mice were similar with that of Control mice at 12 months of age (Control mice: mean ± SD body weight 36.9 g ± 2.8 g and body length 9.7 cm ± .3 cm, Cart-miR-23clus KO mice: 35.5 g ± 2.8 g, 9.6 cm ± .3 cm) and 18 months of age (Control mice: body weight 36.7 g ± 2.5 g and body length 10.2 cm ± .3 cm, Cart-miR-23clus KO mice: 36.8 g ± 5.8 g, 9.8 cm ± .3 cm). Cart-miR-23clus KO mice at 12 months of age exhibited almost normal knee joint tissues including articular cartilage with similar proteoglycan staining, meniscus, synovium, and ScB (Figures 3A,B). At 18 months of age, several Cart-miR-23clus KO mice and Control mice showed reduced Safranin O staining especially in the medial tibial plateau, indicating proteoglycan loss, a roughened articular surface, and fibrillations (Figure 3A). Furthermore, they also showed medial meniscus degeneration, synovitis, and ScB changes with sclerosis in medial tibia (Figure 3A). Although OARSI, meniscus and ScB scores demonstrated more progressive OA-like changes in both mice at 18 months of age than at 12 months of age, there was no significant difference between Cart-miR-23clus KO mice and Control mice at 12 and 18 months of age (Figure 3B). There were no changes in the expression patterns of OA-related markers, ADAMTS5, MMP13, and Type X Collagen between the Control mice and Cart-miR-23clus KO mice (Figures 3C, D).

**FIGURE 4:**
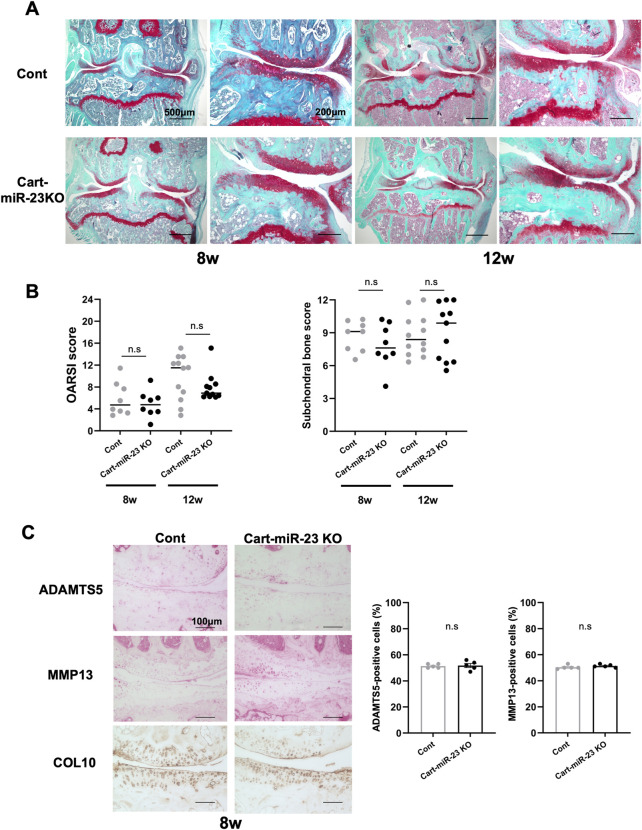
Histopathological evaluation of Knee joint tissues of Cart-miR-23clus KO mice in surgically induced OA model. **(A)** Safranin-O staining in the knee joints of Control and Cart-miR-23clus KO mice in surgically induced OA model. Scale bars: 500 µm and 200 µm. **(B)** The right knee joints of Control and Cart-miR-23clus KO mice after 8 weeks (Control: *n* = 8, Cart-miR-23clus KO: *n* = 8) and 12 weeks (Control: *n* = 12, Cart-miR-23clus KO: *n* = 11) of surgery were assessed by OARSI scoring and ScB scoring system. The data was represented as median. Scoring data was analyzed by Kruskal–Wallis test, then Dunn’s multiple comparison test was applied. **(C)** Knee joints from Control and Cart-miR-23clus KO mice after 8 weeks of surgery were assessed by immunohistochemistry using anti-ADAMTS5, anti-MMP13, and anti-type X Collagen antibodies (*n* = 5 per group). Scale bars: 100 µm. The data represented as mean ± SEM. Comparison of mean values was performed using Welch’s *t*-test. n. s: non-significant difference.

Next, we investigated surgically-induced OA model mice as posttraumatic OA. Both Cart-miR-23clus KO mice and Control mice exhibited mild OA-like changes with proteoglycan loss after 8 weeks of surgery, and the OARSI and ScB scores were not significantly different (Figures 4A, B). After 12 weeks of surgery, both strains of mice exhibited more severe OA-like changes with partial cartilage defects and osteophytes on the medial tibial plateau (Figures 4A,B). OARSI scores indicated more progressive OA-like changes in both mice at 8 weeks and 12 weeks after surgery. However, there was no significant difference between Cart-miR-23clus KO mice and Control mice (Figure 4B). ADAMTS5 and MMP13 expression in articular cartilage did not change in both strains at 8 weeks after surgery (Figure 4C). These results indicated that deficiency of miR-23a/b clusters in chondrocytes does not affect posttraumatic OA severity.

### 3.3 Gene expressions in chondrocytes from miR-23clus KO mice

**FIGURE 5:**
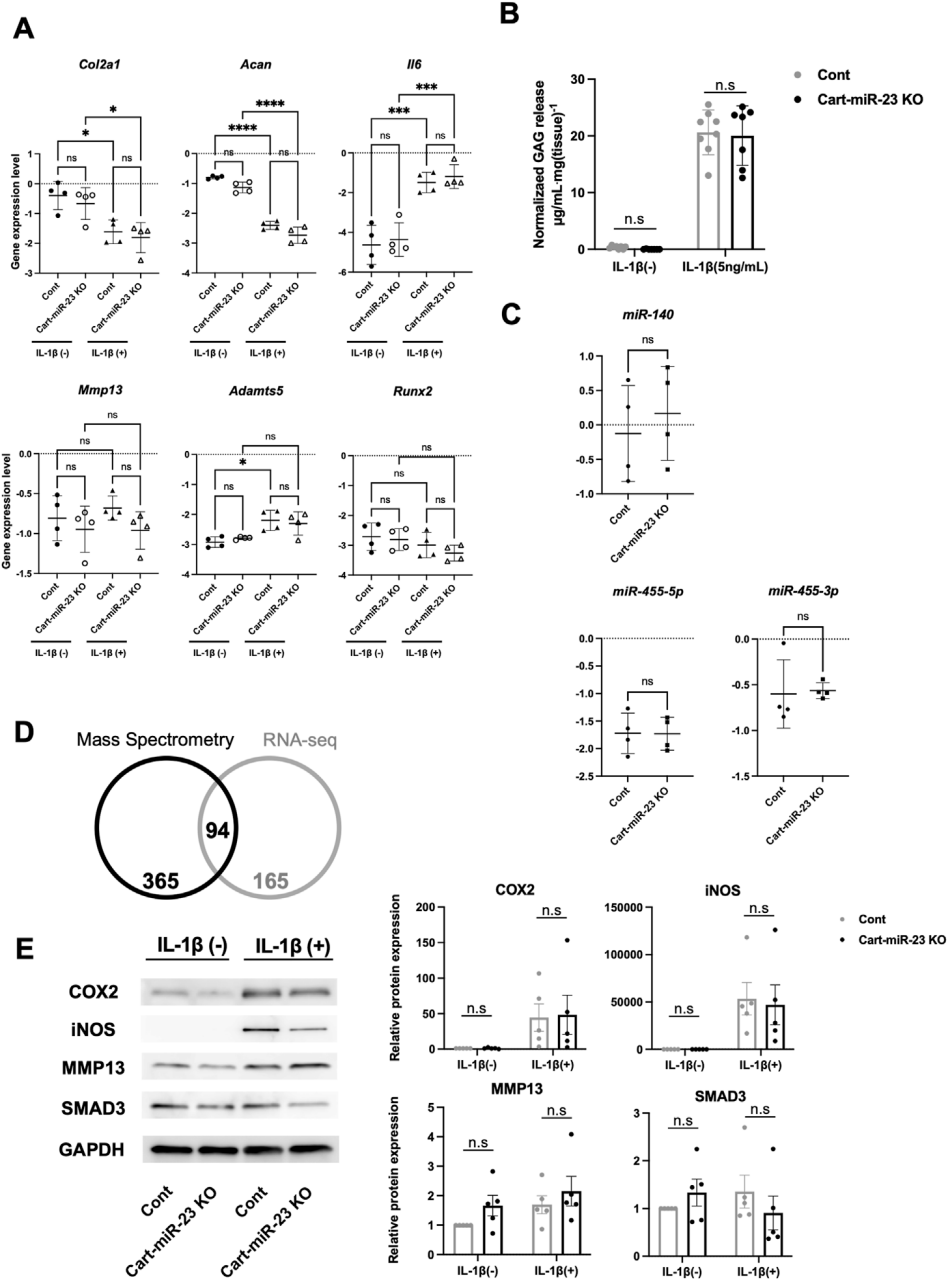
Osteoarthritis related markers in the articular chondrocytes of Cart-miR-23a/b KO mice. **(A)** Real-time PCR was performed to assess the OA-related genes expression in articular chondrocytes with or without IL-1β treatment (1 ng/ml for 24 h) from Control and Cart-miR-23clus KO mice (*n* = 4 each group) at 3 weeks of age. **(B)** Femoral head cartilage explants from Control and Cart-miR-23clus KO mice (Control: *n* = 8, Cart-miR-23clus KO: *n* = 7) cultured with or without IL-1β (5 ng/ml) for 72 h. Proteoglycan release into the conditioned medium from articular cartilage was assayed as the concentration of glycosaminoglycan (GAG). **(C)** Expression of cartilage-related miRNAs in Control and Cart-miR-23clus KO articular chondrocytes by real-time PCR (*n* = 4 each group). **(D)** Venn diagram comparing upregulated proteins and mRNAs (Cart-miR-23clus KO/wild-type mice) in proteome analysis and transcriptome analysis. **(E)** Western blotting of NF-κB-dependent proteins in Control and Cart-miR-23clus KO articular chondrocytes (*n* = 5 each group) with or without IL-1β treatment. All data represented as mean ± SEM. Comparison of gene expression **(A)**, GAG release **(B)** and protein expression **(E)** were performed using one-way ANOVA Sidak’s multiple comparisons test. Comparison of mean values **(C)** was performed using Welch’s *t*-test. *p < .05, **p < .01, ***p < .001, ****p < .0001. n. s: non-significant difference.

Furthermore, we investigated OA-related genes expression and the responses to IL-1β-treatment in chondrocytes from Cart-miR-23clus KO mice at 3 weeks of age. IL-1β stimulation induced a significant increase in the expression of *Adamts5* and inflammatory *Il6* and a significant decrease in the expression of *Col2a1* and *Acan* in chondrocytes from Control mice and/or Cart-miR-23clus KO mice (Figure 5A). However, there were no significant differences between Control mice and Cart-miR-23clus KO mice (Figure 5A). Furthermore, GAG loss levels from articular cartilage of the Cart-miR-23clus mice were not significantly different from that of the Control mice (Figure 5B). Thus, the responses to IL-1β in Cart-miR-23clus KO chondrocytes were similar with Control chondrocytes. The expression of cartilage-related miRNAs, such as miR-140 and miR-455, had no significant changes between Control and Cart-miR-23clus KO chondrocytes (Figure 5C).

### 3.4 Target genes of miR-23 a/b clusters

To investigate mRNA and protein level of miR-23a/b clusters target candidates, we performed multiomics analysis using wild-type chondrocytes and Cart-miR-23clus KO chondrocytes. Four hundred and fifty-nine target candidate genes for miR-23a/b clusters (2-fold increase for Cart-miR-23clus KO chondrocytes compared to Control) were identified by proteomics analysis, and 94 genes were commonly upregulated in mRNA and protein level (Figure 5D; Supplementary Table S5). To further investigate the altered biological processes as a result of miR-23a/b clusters-deletion in chondrocytes, gene ontology (GO) analysis was performed. The upregulated genes were involved in biological processes such as cell adhesion, and downregulated genes were involved in ossification (Supplementary Figures S2, S3). Furthermore, although previous reported target genes were not included in the 94 target candidate genes, to validate NF-κB-dependent genes and *Smad3* as target genes of miR-23a/b clusters, we performed Western Blotting analysis using Control and Cart-miR-23clus KO chondrocytes with or without IL-1β treatment. Although the protein levels of COX2, iNOS and MMP13 were upregulated in Control and miR-23clus KO chondrocytes with IL-1β treatment, there were no significant changes in their upregulation in miR-23clus KO chondrocytes compared with Control chondrocytes (Figure 5E). SMAD3 had no significant difference between Control and miR-23clus KO chondrocytes.

### 3.5 Global miR-23a/b clusters loss-of-function model

**FIGURE 6:**
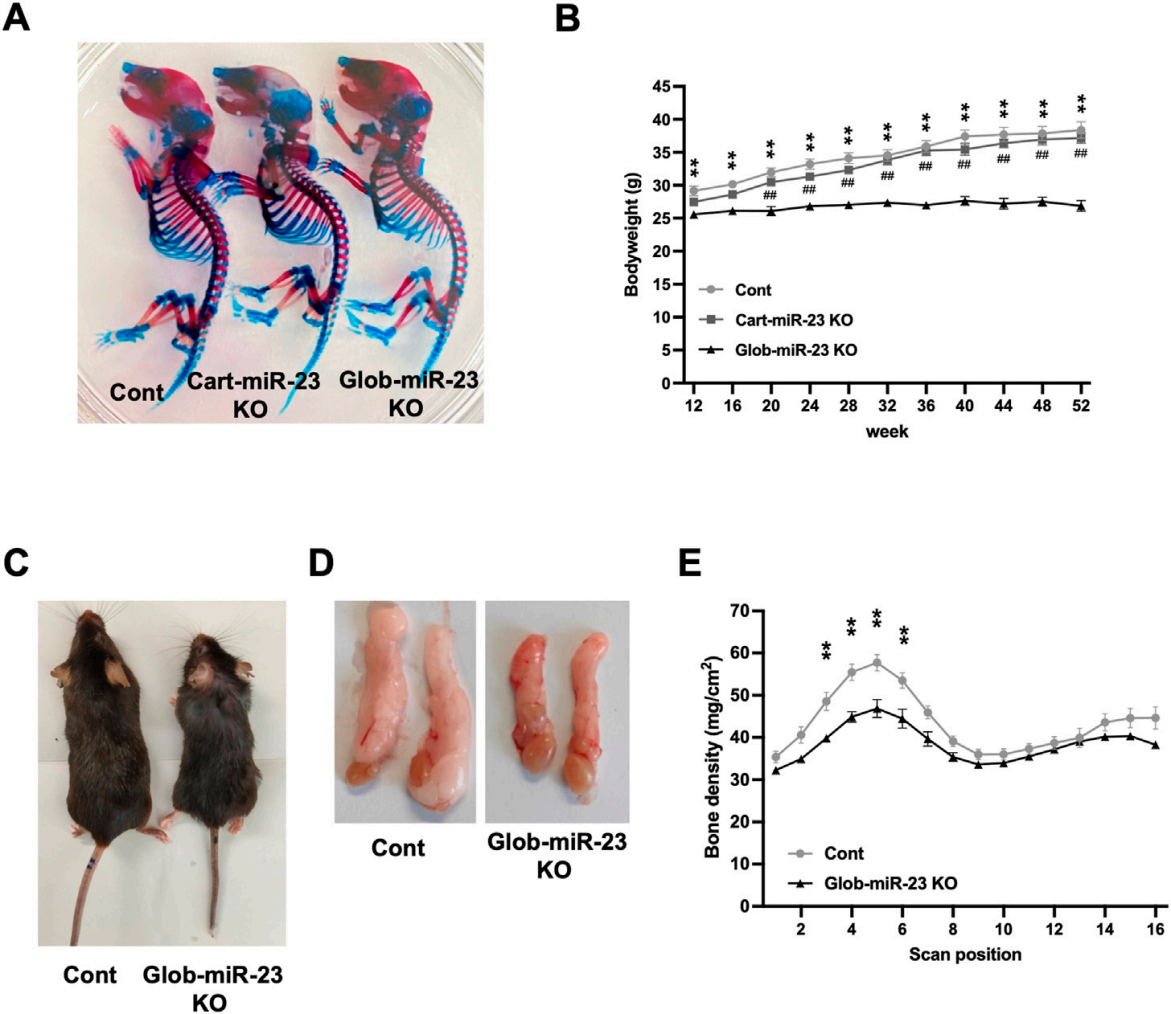
Growth retardation and accelerating aging-like phenotype in Glob-miR-23clus KO mice. **(A)** Skeletal preparation with Alcian blue/Alizarin red staining of Control, Cart-miR-23clus KO, and Glob-miR-23clus KO mice at postnatal day 0. **(B)** Bodyweight during aging. Glob-miR-23clus KO mice exhibited growth retardation during aging. Control: *n* = 11, Cart-miR-23clus KO mice: *n* = 7, Glob-miR-23clus KO mice: *n* = 13. **(C,D)** Representative images of whole body and epididymal adipose tissue in Control and Glob-miR-23clus KO mice at 12 months of age. **(E)** Bone mineral density in Control and Glob-miR-23clus KO mice at 12 months of age (*n* = 7 each group). All data was represented as mean ± SEM. The data **(B,E)** was analyzed by two-way ANOVA; **p < .01:Glob-miR-23clus KO vs. Control, ^##^ *p* < .01:Glob-miR-23clus KO vs. Cart-miR-23clus KO.

**FIGURE 7:**
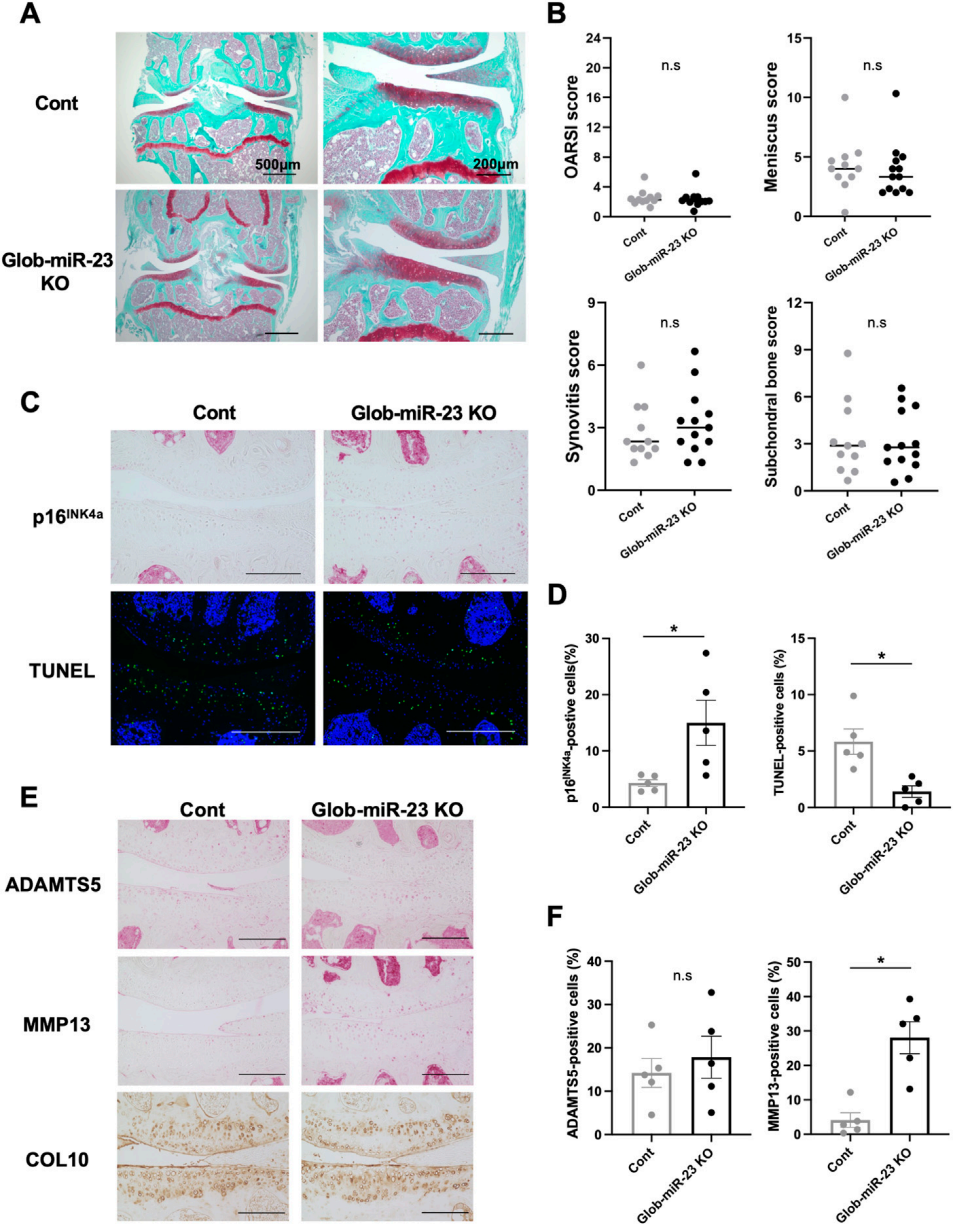
Histopathological evaluation of Knee joint tissues in Glob-miR-23clus KO mice with aging. **(A)** Safranin O staining of the knee joints of Control and Glob-miR-23clus KO mice at 12 months of age. Scale bars: 500 µm and 200 µm. **(B)** The right knee joints of Control and Glob-miR-23clus KO mice at 12 µmmonths of age (Control: *n* = 11, Glob-miR-23clus KO: *n* = 13) were assessed by OARSI scoring, meniscus scoring, synovitis scoring and ScB scoring system. The data was represented as median. Comparison of scoring data was performed by Mann-Whitney *U* test. **(C,D)** Knee joints from Control and Glob-miR-23clus KO mice were assessed by immunohistochemistry using anti-16^INK4a^ antibodies, and TUNEL staining (*n* = 5 per group). Scale bars: 200 µm. **(E,F)** Immunohistochemistry (ADAMTS5, MMP13, and type X Collagen) in articular cartilage of knee joints in Control and Glob-miR-23clus KO mice (*n* = 5 per group). Scale bars: 200 µm. All data was represented as mean ± SEM. Comparison of mean values was performed by Welch’s *t*-test; *p < .05. n. s: non-significant difference.

Furthermore, investigating the role of miR-23a/b clusters in tissues other than cartilage is also important for the skeletal development and the pathogenesis of OA as whole joint or systemic disease. Exosomal miRNAs, which are miRNAs existing in EVs such as exosomes, recently have attracted increasing attention because of their relation to the pathogenesis of various diseases and as newly mediators of tissue-to-tissue/cell-to-cell communication (Valadi et al., 2007; Mori et al., 2019). Thus, to further define the function of miR-23a/b clusters by global deletion including cellular and exosomal miRNA, we evaluated Glob-miR-23clus KO mice with aging. Glob-miR-23clus KO mice, in which miR-23a/b clusters were deleted under the control of the synthetic CAG promoter, exhibited the downregulation of ubiquitous miR-23a/b clusters expression (Supplementary Figure S4). Exosomal miR-23a/b clusters, miR23a, miR-23b, and miR-24 were undetected in serum of Glob-miR-23clus KO mice. miR-27a and miR-27b were undetected in the serum of Control and Glob-miR-23clus KO mice. Skeletal development in Glob-miR-23clus KO mice at postnatal day 0 appeared grossly normal compared with Control mice (Figure 6A). The bodyweight of Glob-miR-23clus KO mice was significantly lower than Control mice and Cart-miR-23clus KO mice throughout aging until 12 months of age (Figure 6B). Their body size and adipose tissue volume were significantly lower than Control mice at 12 months of age (Figures 6C, D). Furthermore, Glob-miR-23clus KO mice exhibited low bone density (Figure 6E). However, Glob-miR-23clus KO mice did not exhibit knee joints with severe OA-like changes at 12 months of age, each score had no significant difference between Control mice and Glob-miR-23clus KO mice (Figures 7A, B). Although the number of cells positive for p16^INK4a^, a marker of senescent cells, were significantly increased in articular cartilage of Glob-miR-23clus KO mice, TUNEL-positive chondrocytes in superficial and middle layer were decreased in Glob-miR-23clus KO mice (Figures 7C, D). Type X Collagen and ADAMTS5 had no changes between articular cartilage of Control mice and Glob-miR-23clus KO mice (Figures 7E, F). MMP13 was significantly increased in articular cartilage of Glob-miR-23clus KO mice (Figures 7E, F). However, Glob-miR-23clus KO mice also did not exhibit acceleration of OA development.

## 4 Discussion

In the present study, we determined the role of miR-23a/b clusters, which are highly expressed in cartilage, in the pathogenesis of OA using two OA models in miR-23a/b clusters KO mice. Among miR-23a/b clusters, miR-23a, miR-23b and miR-27b have been suggested to have a role in maintaining articular cartilage state and anti-inflammation by *in vitro* studies (Akhtar et al., 2010; Hu et al., 2017; Zhou et al., 2017; Xu et al., 2018; Lv et al., 2020; Zhou et al., 2021). On the other hand, several reports suggest that miR-23a and miR-23b contribute to OA progression (Kang et al., 2016; Guo et al., 2018; Zhao et al., 2019). Thus, the role of each miRNA in miR-23a/b clusters is still controversial in OA pathogenesis. One of the reasons is that the deletion of a single miRNA in miRNA cluster may be compensated for by functionally redundant another miRNA in cluster. The present study demonstrated that miR-23 a/b clusters-deletion have no critical role in the severity of OA with aging and following surgical trauma. This suggests that miR-23a/b clusters, composed of miR-23a, miR-23b, miR-24, miR-27a and miR-27b is not essential for cartilage homeostasis.

MiR-23a/b clusters were highly expressed in cartilage. Among them, miR-23a, miR-24, and miR-27a were significantly downregulated in OA chondrocytes. However, miR-23clus KO mice did not accelerate the severity of OA. In the screening of target genes for miR-23a/b clusters using multiomics analysis, 94 candidate genes were listed. The upregulated genes and proteins in miR-23clus KO chondrocytes might not only be candidate target genes for miR-23a/b clusters but also altered secondarily to compensate for the functionally complement genes due to the deficiency of miR-23a/b clusters in chondrocytes. However, the upregulated genes and proteins mean that they were not essentially associated with OA pathogenesis as shown by the phenotype of miR-23clus KO mice. Although we listed cartilage-miRNAs (Supplementary Table S4), functioning coordinately with other miRNAs might be a potential explanation for the lack of OA-like phenotype of miR-23a/b clusters deficiency in cartilage. Indeed, the expression level of OA-related genes might be coordinately regulated by multiple miRNAs that are highly expressed in cartilage. We can find a target gene for multiple miRNAs like this in OA-related genes from the database, TargetScan 8.0 (https://www.targetscan.org/vert_80/). For example, we found that cartilage-degrading enzyme, *Adamts5*, is a common target gene for miR-23a, miR-27a and miR-140 (Supplementary Table S6). Thus, the phenotype of double KO mice of miR-23a/b clusters and other cartilage-miRNA might be a potential explanation for fine-tuning the expression level of common target genes and causing OA-like pathological conditions. Currently, we are working on to further clarify the reason for the lack of the phenotype in the present study. In addition, previous reported target genes of miR-23, miR-24 and miR-27a such as *Runx2*, murine double minute 4 (*Mdm4*), *Smad3*, *Leptin* and *Mmp13* (Zhang et al., 2011; Zhang et al., 2012; Kang et al., 2016; Zhou et al., 2017; Li et al., 2018; Zhao et al., 2019; Xu et al., 2021) were not included among 94 candidate genes. The identification of target gene for miRNAs depends on the kinds of cell type, and endogenous target gene expression level under various conditions such as aging and inflammation in *in vitro* and *in vivo*. Furthermore, many target genes for miR-23a/b clusters have been identified by *in vitro* model using excessive miR-23a/b mimic or inhibitor. These factors might be one of the reasons.

Aging and age-related diseases have been characterized by the accumulation of senescent cells in various tissues including cartilage and are associated with age-related pathogenesis (Baker et al., 2016; Farr et al., 2017; Jeon et al., 2017). Glob-miR-23clus KO mice exhibited low bone density at 12 months of age. Although miR-24 is a negative regulator of the senescence marker p16^INK4a^ (Philipot et al., 2014), Glob-miR-23clus KO mice did not show increased severity of OA. This lack of an OA phenotype is despite the fact that we observed some molecular and cellular changes (increased MMP13 and p16^INK4a^-positive senescent cells in cartilage) in the Glob-miR-23clus KO mice with aging. A recent study reported that p16^INK4a^ expression is a biomarker of dysfunctional chondrocytes but does not cause OA (Diekman et al., 2018). Our results may support that, and/or OA may develop at a later stage compared with aging-like phenotype such as osteopenia. Furthermore, the present study indicated that the deficiency of miR-23 a/b clusters in other tissues (other cells) except cartilage (chondrocytes), and in body fluids may have caused the impaired growth including skeletal growth with aging. Previously, tissue-specific miR-23a/b clusters deficient mice, such as vascular endothelial cells- and muscle-specific miR-23a/b clusters deficient mice, exhibit normal growth and have no dramatic effects on tissue or cell functions (Oikawa et al., 2018; Lee et al., 2019). In bone, miR-23a/b clusters inhibit osteogenesis by targeting *Runx2*, *Satb2*, *Sp7* (*Osterix*), and *Bmpr1b in vitro* (Hassan et al., 2010; Peng et al., 2017; Godfrey et al., 2018; Zhang et al., 2019). Global-miR-23a transgenic (Tg) mice revealed a limited role in bone formation and maintenance (Park et al., 2015). Osteoblast-specific miR-23a cluster Tg mice exhibit low bone mass through targeting of *Prdm16* (Zeng et al., 2017). On the other hand, male and female miR-23a cluster knockdown mice (homozygous miR-23a Cl^*ZIP*^ mice) died postnatally, and female miR-23a cluster knockdown mice (hetero zygous miR-23a Cl^*ZIP*^ mice) had increase bone density (Godfrey et al., 2018). The phenotype due to the difference of sex in miR-23clus KO mice should further be examined since only male mice were investigated in the present study. Furthermore, recently, exosomal miRNAs have attracted increasing attention because of their relation to the pathogenesis of various diseases as new mediators of tissue-to-tissue/cell-to-cell communication (Valadi et al., 2007; Mori et al., 2019). miR-23a from osteoclast-derived EVs suppress osteogenesis by targeting *Runx2* (Yang et al., 2020). From previous studies and the present study, however, aging-like phenotype with osteopenia in Glob-miR-23clus KO mice might be due to the impairment of endocrine and nervous system rather than derived from skeletal system-related cells such as chondrocytes, muscle cells, osteoblasts, osteocytes and osteoclasts. Furthermore, miR-23a/b clusters KO mice show decreased bone marrow cellularity and hematopoietic stem cell populations (Kurkewich et al., 2018). Thus, we should further examine what functions of miR-23a/b clusters including exosomal miRNAs is derived from which cells, and their target genes in order to reveal the mechanism of accelerated aging-like phenotype such as osteopenia in Glob-miR-23clus KO mice. These future results will open a new insight in aging mechanisms through the miR-23 a/b clusters.

Together, miR-23a and -23b clusters were highly expressed in various tissues including cartilage. However, loss-of-function studies using cartilage-specific- and global-miR-23clus KO mice demonstrated that the biomedical function of miR-23 a/b clusters in chondrocytes is not essential for OA pathogenesis.

## Data Availability

The datasets presented in this study can be found in online repositories. The names of the repository/repositories and accession number(s) can be found in the article/Supplementary Material.
