# Supplementary material for: miR-23a/b clusters are not essential for the pathogenesis of osteoarthritis in mouse aging and post-traumatic models
Source: Front Cell Dev Biol. 2023 Jan 4;10:1043259. doi: 10.3389/fcell.2022.1043259 (PMC9846268; doi:10.3389/fcell.2022.1043259)
Supplement: Supplementary file 1 [file DataSheet1.PDF]

*Supplementary Material*

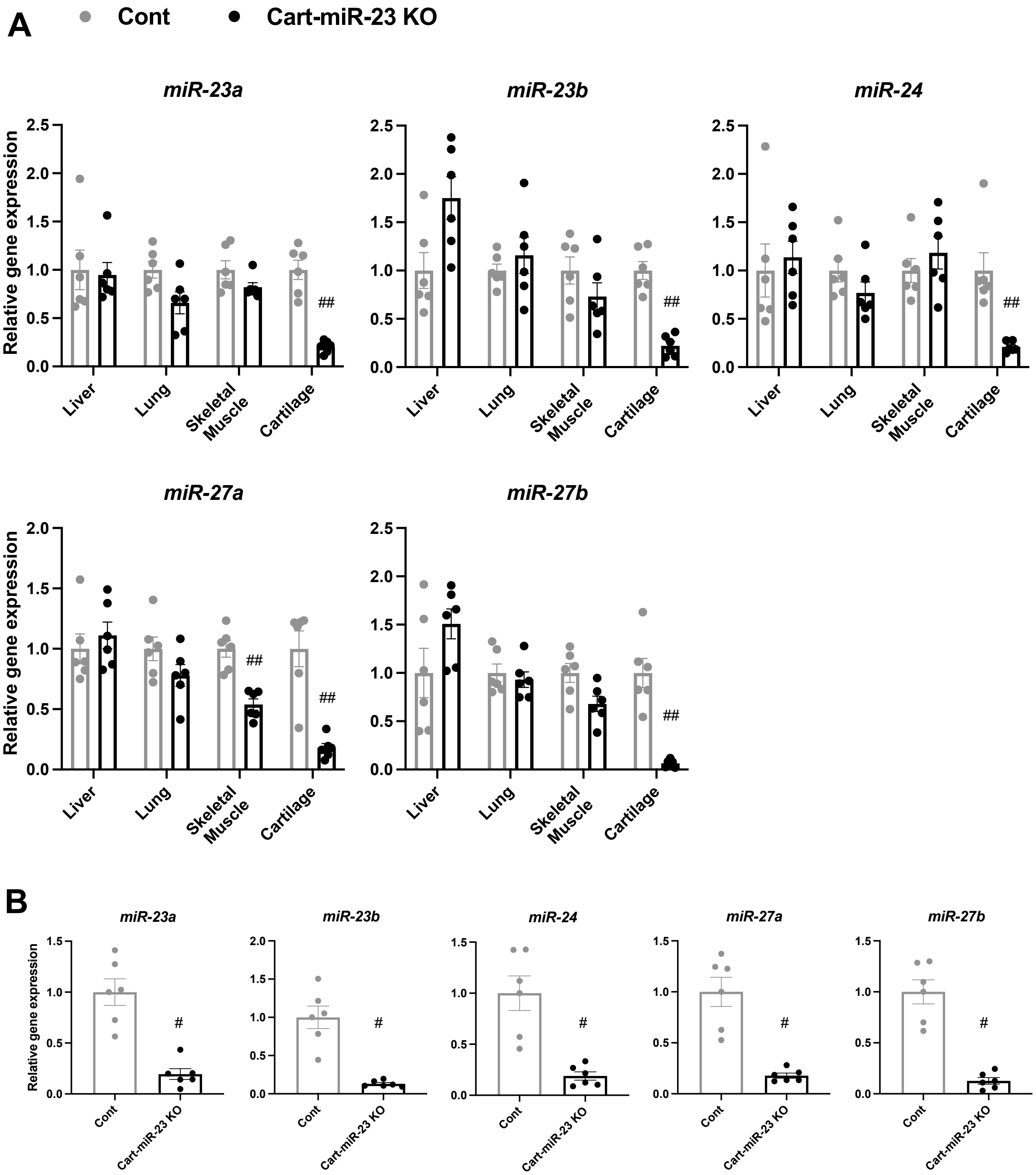

**Fig. S1 Validation of cartilage-specific miR-23a/b clusters deficiency in Cart-miR-23clus KO mice.**

**A**) Real-time analysis showed the expression of miR-23a/b clusters in liver, lung, skeletal muscle, and articular cartilage from Control and Cart-miR-23clus KO mice at 4 weeks of age. **B**) The expression of miR-23a/b clusters in articular chondrocytes from Control and Cart-miR-23clus KO mice. All data are represented as mean ± SEM. Comparisons of expression level were performed by Mann-Whitney tests in each tissue. Holm-Sidak corrections were applied to correct for multiple comparisons. $^{\#}P<0.05$, $^{\#\#}P<0.01$. n=6 per group.

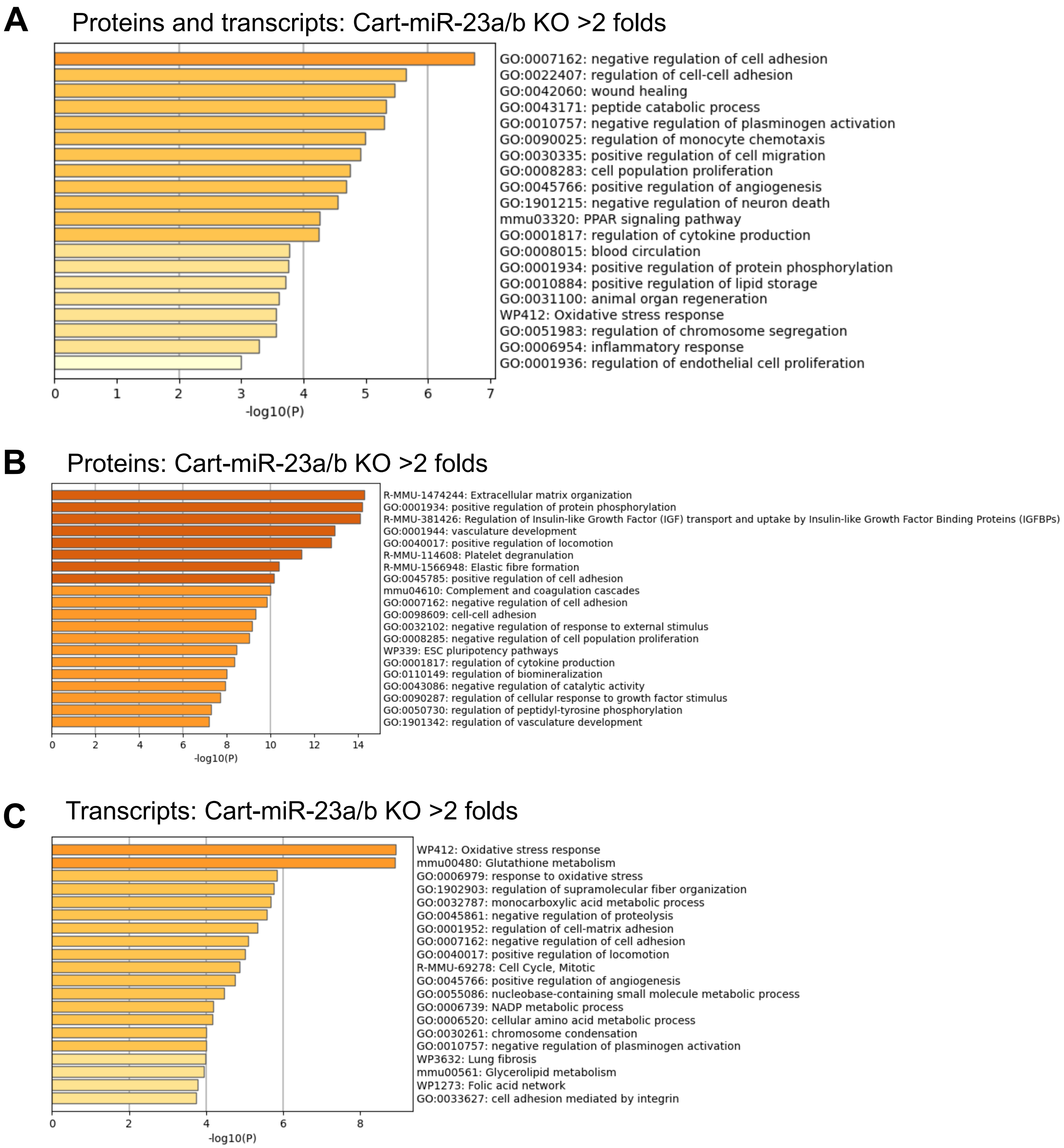


**Fig. S2** Top 20 gene ontology enrichment analysis terms for the genes which **A)** proteins and transcripts are both have been upregulated (>2 folds) **B)** proteins have been upregulated (>2 folds) **C)** transcripts have been upregulated (>2 folds) in articular chondrocytes from Cart-miR-23a/b KO mouse compared to Control mouse.

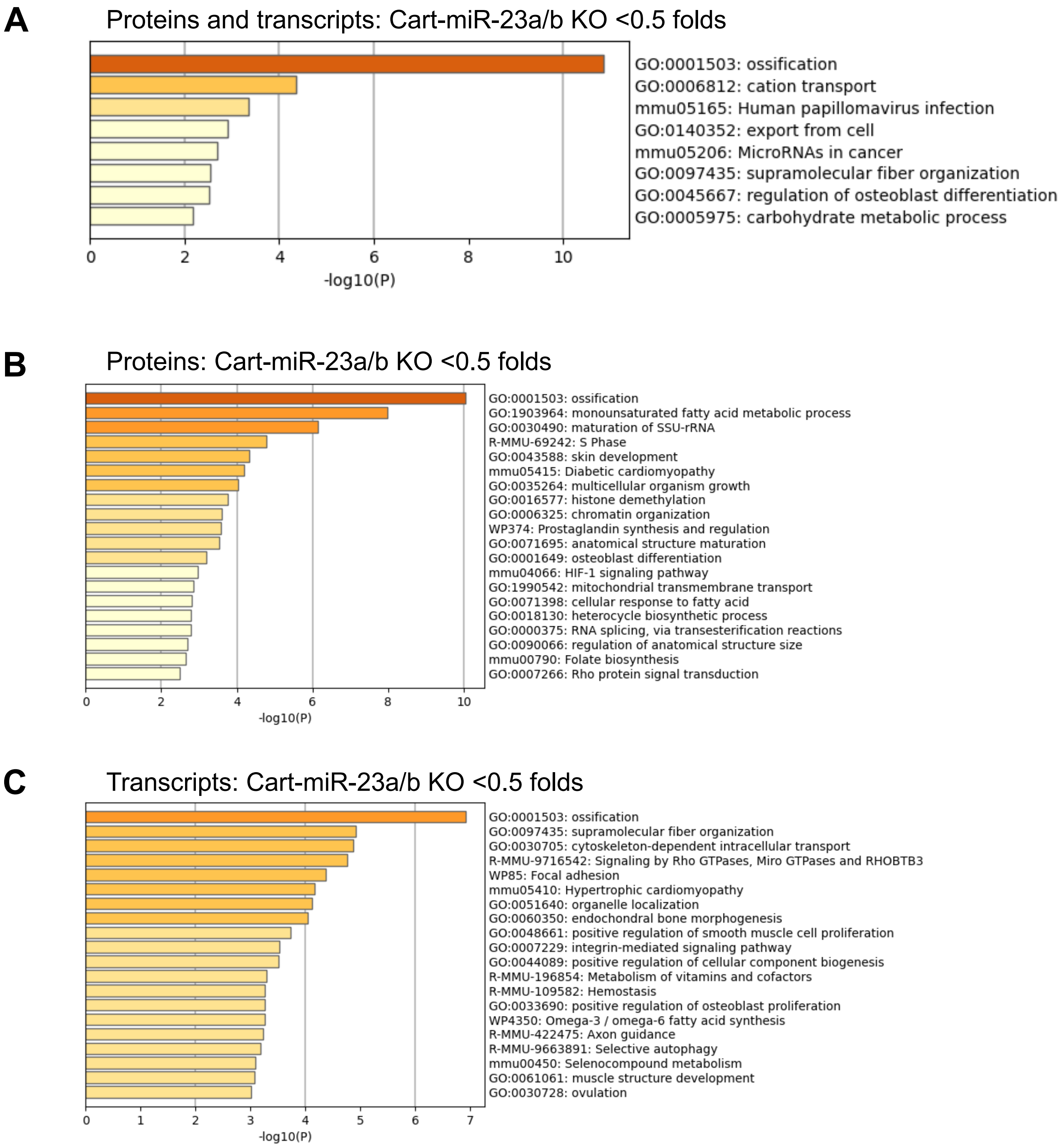


**Fig. S3** Top gene ontology enrichment analysis terms for the genes which **A)** proteins and transcripts are both have been downregulated (<0.5 folds) **B)** proteins have been downregulated (<0.5 folds) **C)** transcripts have been downregulated (<0.5 folds) in articular chondrocytes from Cart-miR-23a/b KO mouse compared to Control mouse.

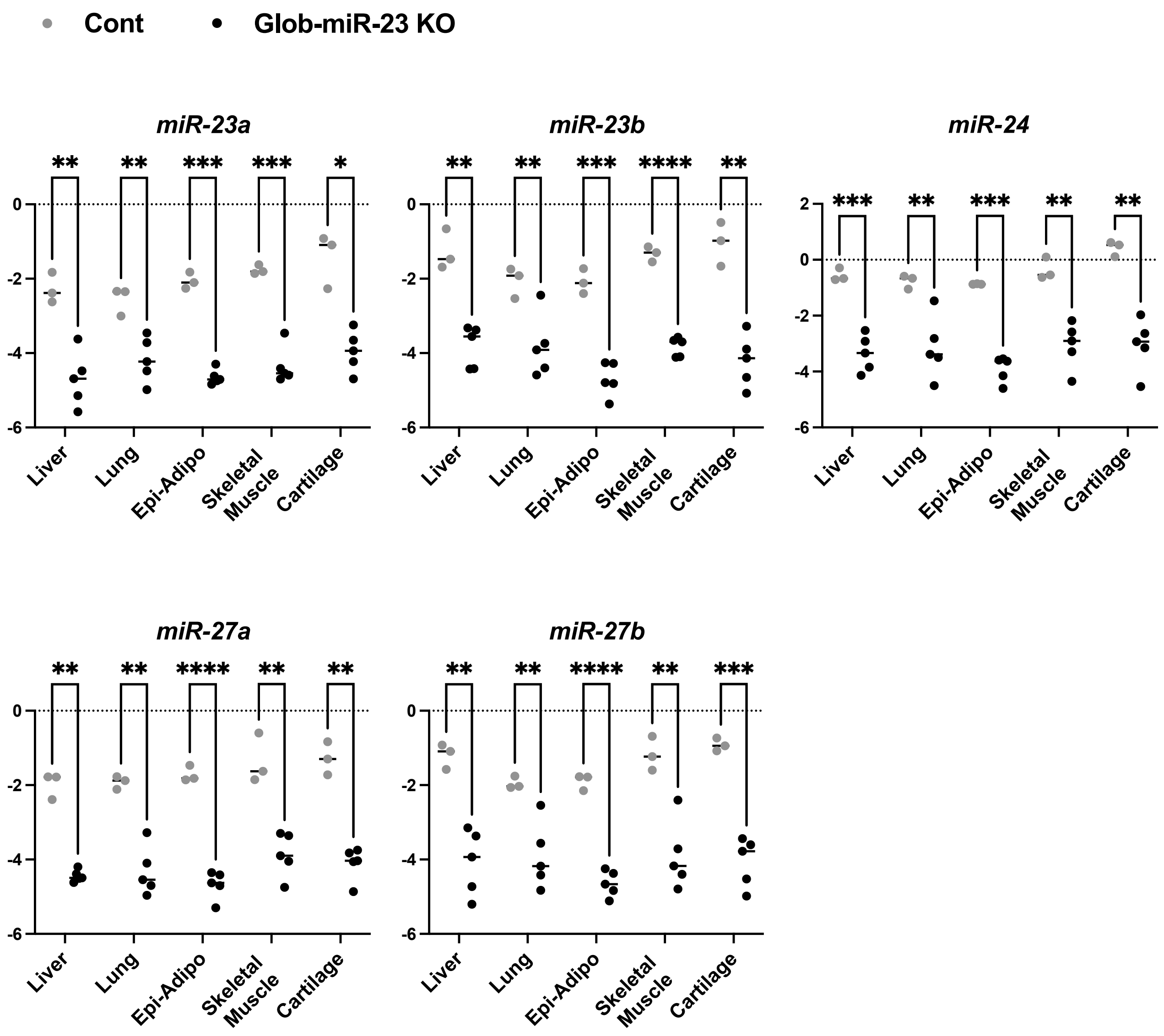


**Fig. S4 Validation of miR-23a/b clusters deficiency in various tissues of Glob-miR-23clus KO mice.**

Real-time PCR analysis showed the expression of miR-23a/b clusters in liver, lung, epididymal adipose tissue, skeletal muscle, and articular cartilage from Control (n=3) and Glob-miR-23clus KO mice (n=5) at 4 weeks of age. All data are represented as mean ± SEM. Comparisons of expression level were performed by multiple welch test. *P<0.05, **P<0.01, ***P<0.001, ****P<0.0001.

**Table. S1**

| | Primer sequence |
|---|---|
| ***miR-23a cluster**** | F: GCTCCAACCTTCCTACGGATCGATGC |
| | R-1: CCTGCCTCTACCTCTGGAGTCTAGGA |
| | R-2: GTGGTGCAGCTGGTATTCCCAAACTC |
| ***miR-23b cluster**** | F: TGCCCCCTGAGTGAGCAAATCC |
| | R-1: TGGCTTGCCTGTGACCAAGCAT |
| | R-2: GGTGTCCTTCATTGAATGACTGCC |
| ***Cre recombinase*** | F: GCATTACCGGTCGATGCAACGAGTGATGAG |
| | R: GAGTGAACGAACCTGGTCGAAATCAGTGCG |

*For the genotyping of the miR-23a or miR-23b cluster, primer-F and primer-R-1 were used for the Cart:miR-23clusKO mice and the combinations of primer-F with primer-R-1 and primer-F with primer-R-2 were used for the Glob-miR-23clusKO mice.

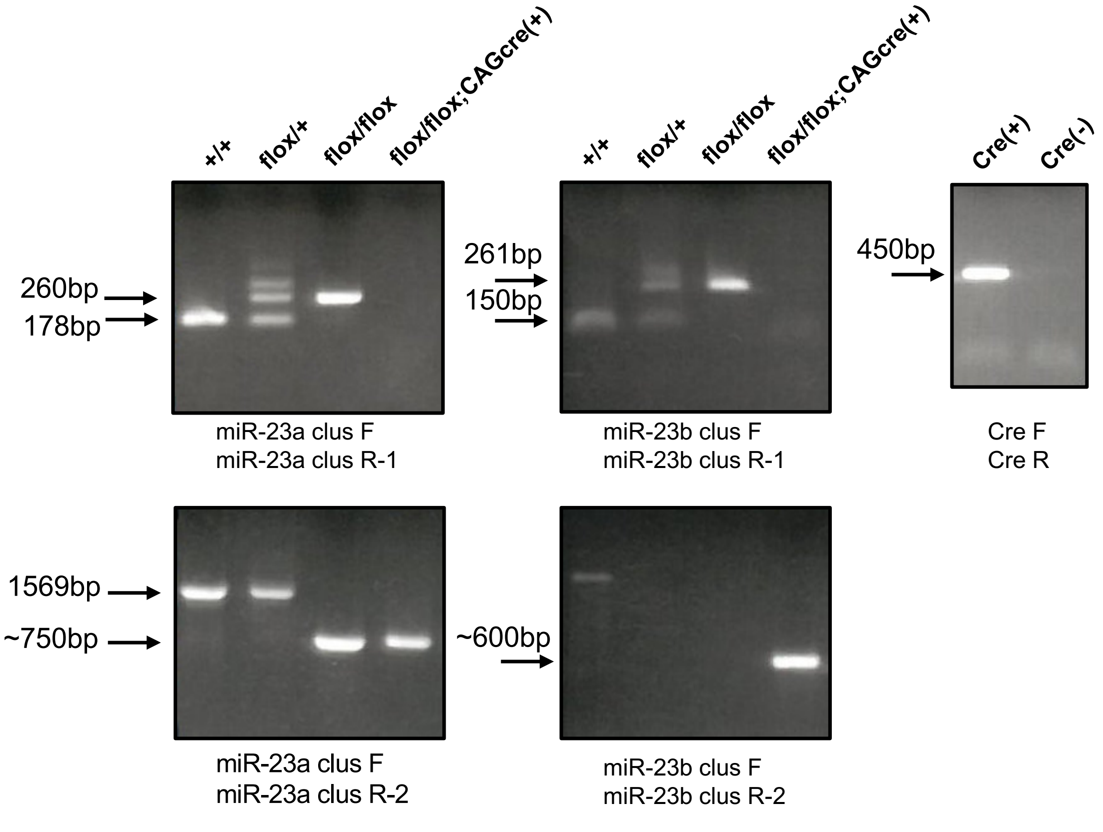

**Table. S2**

| Gene Name | Assay ID |
|---|---|
| ***Col2a1*** | Mm01309565m1 |
| ***Acan*** | Mm00545807m1 |
| ***Mmp13*** | Mm00439491m1 |
| ***Adamts5*** | Mm00478620m1 |
| ***Il6*** | Mm00446190m1 |
| ***Runx2*** | Mm00446190m1 |
| ***Gapdh*** | Mm99999915g1 |
| ***miR-23a-3p*** | RT/TM000399 |
| ***miR-23b-3p*** | RT/TM000400 |
| ***miR-24-3p*** | RT/TM000402 |
| ***miR-27a-3p*** | RT/TM000408 |
| ***miR-27b-3p*** | RT/TM000409 |
| ***miR-140-5p*** | RT/TM000462 |
| ***miR-455-3p*** | RT/TM002244 |
| ***miR-455-5p*** | RT/TM001280 |
| ***U6 snRNA*** | RT/TM001973 |

Real time PCR was performed with the TaqMan Gene Expression Assay probes

**Table. S3**

| Antibody | Catalog Number | Manufacturer |
|---|---|---|
| **COX2 (mouse, monoclonal)** | sc-376861 | Santa Cruz Biotechnology |
| **iNOS (rabbit, polyclonal)** | ab15323 | Abcam |
| **MMP13 (mouse, monoclonal)** | MA5-14238 | Thermo Fisher Scientific |
| **SMAD3 (rabbit, monoclonal)** | ab40854 | Abcam |
| **GAPDH (mouse, monoclonal)** | 015-25473 | FUJIFILM Wako |

**Table. S4**

| | Chondrocyte | | Cho-EVs | |
|---|---|---|---|---|
| **microRNA** | **Rank** | **Normalized Counts** | **Rank** | **Normalized Counts** |
| hsa-let-7a-5p | 1 | 47048.64 | 5 | 12162.12 |
| hsa-miR-125b-5p | 2 | 35194.21 | 2 | 35106.55 |
| hsa-miR-4454 | 3 | 26155.59 | 1 | 49397.53 |
| hsa-let-7b-5p | 4 | 21205.61 | 12 | 4336.57 |
| hsa-miR-21-5p | 5 | 20252.24 | 3 | 17950.79 |
| **hsa-miR-23a-3p** | **6** | **13823.68** | **4** | **15808.79** |
| hsa-miR-29b-3p | 7 | 13301.61 | 14 | 3962.05 |
| hsa-let-7g-5p | 8 | 7865.05 | 15 | 3193.30 |
| hsa-miR-100-5p | 9 | 7533.65 | 8 | 5939.79 |
| hsa-miR-99a-5p | 10 | 5992.56 | 12 | 4336.57 |
| hsa-miR-720 | 11 | 5370.90 | 6 | 7096.21 |
| hsa-miR-221-3p | 12 | 5224.52 | 24 | 2293.13 |
| hsa-miR-199a-3p+hsa-miR-199b-3p | 13 | 4641.19 | 9 | 5210.46 |
| hsa-miR-222-3p | 14 | 4160.89 | 11 | 4737.38 |
| **hsa-miR-27b-3p** | **15** | **4019.85** | **18** | **2904.19** |
| hsa-miR-15b-5p | 16 | 3885.41 | 20 | 2509.96 |
| **hsa-miR-23b-3p** | **17** | **3869.70** | **16** | **3134.16** |
| hsa-miR-15a-5p | 18 | 3536.10 | 22 | 2345.69 |
| hsa-miR-22-3p | 19 | 3050.78 | 21 | 2385.12 |
| hsa-miR-125a-5p | 20 | 2810.47 | 32 | 1373.25 |
| hsa-miR-191-5p | 21 | 2556.97 | 19 | 2825.34 |
| hsa-miR-374a-5p | 22 | 2547.24 | 26 | 1951.46 |
| hsa-miR-26a-5p | 23 | 2530.90 | 42 | 762.19 |
| hsa-miR-148a-3p | 24 | 2329.55 | 23 | 2339.12 |
| hsa-miR-199a-5p | 25 | 2218.98 | 27 | 1866.04 |
| hsa-miR-376a-3p | 26 | 1856.48 | 17 | 3055.31 |
| hsa-miR-29a-3p | 27 | 1789.57 | 29 | 1662.35 |
| hsa-miR-16-5p | 28 | 1717.63 | 28 | 1714.92 |
| hsa-miR-337-5p | 29 | 1683.71 | 31 | 1438.95 |
| **hsa-miR-140-5p** | **30** | **1445.29** | **36** | **1136.71** |
| hsa-let-7i-5p | 31 | 1313.67 | 46 | 670.20 |
| hsa-let-7c | 32 | 1133.99 | 58 | 473.08 |
| hsa-miR-34a-5p | 33 | 1117.03 | 51 | 525.65 |
| hsa-miR-376c | 34 | 1084.04 | 33 | 1327.25 |
| hsa-miR-361-5p | 35 | 1059.54 | 40 | 834.46 |
| hsa-miR-199b-5p | 36 | 1042.58 | 52 | 519.07 |
| hsa-let-7e-5p | 37 | 1042.27 | 70 | 341.67 |
| hsa-miR-377-3p | 38 | 882.69 | 30 | 1550.65 |
| hsa-let-7f-5p | 39 | 838.71 | 94 | 243.11 |
| hsa-miR-130a-3p | 40 | 809.50 | 37 | 1005.30 |
| hsa-miR-382-5p | 41 | 718.40 | 56 | 479.65 |
| hsa-miR-155-5p | 42 | 712.12 | 778 | 6.57 |
| hsa-miR-106a-5p+hsa-miR-17-5p | 43 | 681.96 | 71 | 335.10 |
| hsa-miR-127-3p | 44 | 626.99 | 43 | 729.33 |
| **hsa-miR-24-3p** | **45** | **626.36** | **47** | **663.63** |
| … | … | … | … | … |
| **hsa-miR-27a-3p** | **98** | **167.74** | **126** | **164.26** |

Cho-EVs: Chondrocyte-derived extracellular vehicles

## Table. S5

| | Mass Spectrometry (Identified Peptide Count) | | | RNA-seq (TPM) | | |
|---|---|---|---|---|---|---|
| Gene name | Cont | Cart-miR-23 KO | Fold Change | Cont | Cart-miR-23 KO | Fold Change |
| Hmox1 | 447277000 | 1479628000 | 3.31 | 53.07 | 153.81 | 2.90 |
| Serpine2 | 394245800 | 792757500 | 2.01 | 370.31 | 954.20 | 2.58 |
| Serpinb1a | 235063600 | 512885100 | 2.18 | 17.47 | 45.60 | 2.61 |
| Gpnmb | 184437900 | 429767000 | 2.33 | 125.39 | 343.42 | 2.74 |
| Maoa | 170023800 | 492563200 | 2.90 | 4.59 | 29.34 | 6.39 |
| Plin4 | 148102900 | 420612600 | 2.84 | 12.81 | 45.97 | 3.59 |
| Tpm2 | 136333500 | 339893200 | 2.49 | 61.33 | 124.83 | 2.04 |
| Tagln | 125071800 | 769259900 | 6.15 | 1.80 | 24.45 | 13.58 |
| Ass1 | 73640130 | 196621700 | 2.67 | 2.82 | 12.43 | 4.40 |
| Erap1 | 70216530 | 141026100 | 2.01 | 2.88 | 7.47 | 2.59 |
| Aldh1a1 | 68154590 | 239258900 | 3.51 | 99.69 | 277.79 | 2.79 |
| Fabp4 | 66201750 | 245077400 | 3.70 | 60.89 | 262.29 | 4.31 |
| Mttp | 48886180 | 106763900 | 2.18 | 2.42 | 5.58 | 2.31 |
| Anpep | 46278680 | 154543800 | 3.34 | 3.03 | 8.88 | 2.94 |
| Lox | 45187450 | 346526700 | 7.67 | 244.49 | 981.62 | 4.01 |
| Emb | 41188020 | 82608200 | 2.01 | 402.69 | 977.26 | 2.43 |
| Icam1 | 26620030 | 474050000 | 17.81 | 11.73 | 79.41 | 6.77 |
| Pdlim4 | 25021980 | 76676650 | 3.06 | 12.15 | 41.01 | 3.38 |
| C3 | 22629220 | 49426810 | 2.18 | 0.56 | 3.28 | 5.87 |
| Enpep | 22315770 | 59819150 | 2.68 | 0.46 | 2.27 | 4.95 |
| Gfer | 21487370 | 44308620 | 2.06 | 57.62 | 142.16 | 2.47 |
| Serpine1 | 20536510 | 67098690 | 3.27 | 40.05 | 85.40 | 2.13 |
| Synm | 20516480 | 42151550 | 2.05 | 1.35 | 6.74 | 5.00 |
| Il1rn | 20042700 | 60765570 | 3.03 | 8.90 | 40.04 | 4.50 |
| Plpp3 | 15472890 | 66761900 | 4.31 | 21.82 | 52.04 | 2.39 |
| P4ha3 | 14752360 | 43063280 | 2.92 | 38.11 | 81.68 | 2.14 |
| Rufy3 | 10616410 | 29066600 | 2.74 | 4.39 | 8.82 | 2.01 |
| Rrm2 | 8757544 | 37681400 | 4.30 | 13.09 | 37.48 | 2.86 |
| Lgals9 | 7630653 | 17574580 | 2.30 | 4.41 | 11.09 | 2.52 |
| Top2a | 5817056 | 12842100 | 2.21 | 8.98 | 19.58 | 2.18 |
| Ttyh3 | 5485562 | 35086060 | 6.40 | 10.46 | 21.88 | 2.09 |
| Timp1 | 5446187 | 13657780 | 2.51 | 621.13 | 1797.41 | 2.89 |
| Mt2 | 5337880 | 11282930 | 2.11 | 1470.42 | 7069.73 | 4.81 |
| Sorl1 | 5273122 | 15998130 | 3.03 | 5.00 | 10.23 | 2.04 |
| Vcam1 | 5265081 | 33679840 | 6.40 | 7.73 | 24.00 | 3.11 |
| Xdh | 5139618 | 17784530 | 3.46 | 0.54 | 4.17 | 7.71 |
| Atp1b1 | 4760890 | 20713530 | 4.35 | 0.37 | 6.54 | 17.62 |
| Cyp1b1 | 4467622 | 24652690 | 5.52 | 32.66 | 109.35 | 3.35 |
| Tinagl1 | 4294986 | 8662477 | 2.02 | 15.15 | 49.53 | 3.27 |
| Nid1 | 4017366 | 9807333 | 2.44 | 2.83 | 6.57 | 2.32 |
| Eng | 3920848 | 19044830 | 4.86 | 3.49 | 7.33 | 2.10 |
| Cxcl12 | 3743041 | 82994030 | 22.17 | 389.90 | 2831.06 | 7.26 |
| Clu | 3581137 | 24472920 | 6.83 | 122.16 | 321.40 | 2.63 |
| Prss23 | 3410412 | 23391200 | 6.86 | 33.51 | 71.82 | 2.14 |
| Ppif | 3340422 | 16465260 | 4.93 | 3.68 | 13.91 | 3.77 |
| Myoc | 3077883 | 19585150 | 6.36 | 17.54 | 86.38 | 4.93 |
| Hs6st2 | 3016240 | 28088240 | 9.31 | 42.12 | 119.35 | 2.83 |
| Tjp2 | 2679283 | 6709965 | 2.50 | 1.44 | 4.74 | 3.30 |

*(Continue…)*

**Table. S5**

| | Mass Spectrometry (Identified Peptide Count) | | | RNA-seq (TPM) | | |
|---|---|---|---|---|---|---|
| **Gene name** | **Cont** | **Cart-miR-23 KO** | **Fold Change** | **Cont** | **Cart-miR-23 KO** | **Fold Change** |
| Crlf1 | 2508715 | 18624450 | 7.42 | 8.73 | 43.59 | 4.99 |
| Anln | 2236792 | 5140888 | 2.30 | 2.29 | 4.81 | 2.10 |
| Lpl | 2222832 | 15952490 | 7.18 | 78.62 | 158.78 | 2.02 |
| Ccn3 | 2150312 | 14033260 | 6.53 | 9.01 | 56.57 | 6.28 |
| Ephb6 | 2130838 | 6526919 | 3.06 | 6.36 | 17.09 | 2.69 |
| Tdrp | 2066204 | 4313554 | 2.09 | 0.78 | 3.42 | 4.40 |
| Pm20d1 | 1981226 | 7933452 | 4.00 | 0.28 | 0.76 | 2.75 |
| Il3ra | 1862561 | 4298940 | 2.31 | 1.19 | 2.61 | 2.20 |
| Mgst1 | 1576546 | 4471263 | 2.84 | 4.10 | 10.84 | 2.64 |
| Fabp7 | 1540417 | 4058362 | 2.63 | 1.19 | 3.93 | 3.30 |
| Gba2 | 1414731 | 4404064 | 3.11 | 1.08 | 2.68 | 2.48 |
| Nes | 1177270 | 10239480 | 8.70 | 0.73 | 2.41 | 3.30 |
| Postn | 1162173 | 4845063 | 4.17 | 3.07 | 8.10 | 2.64 |
| Lpin1 | 1133115 | 2668860 | 2.36 | 9.38 | 22.18 | 2.37 |
| Ccdc126 | 1089689 | 2845145 | 2.61 | 2.44 | 5.38 | 2.20 |
| Me3 | 1084754 | 2434075 | 2.24 | 0.65 | 1.66 | 2.57 |
| Prg4 | 1061632 | 3226544 | 3.04 | 65.32 | 140.99 | 2.16 |
| Dlk1 | 990338 | 9978799 | 10.08 | 25.79 | 347.08 | 13.46 |
| Ggt5 | 948152 | 5120349 | 5.40 | 2.33 | 6.60 | 2.83 |
| Kif1a | 939535 | 6737272 | 7.17 | 0.93 | 2.18 | 2.34 |
| Mustn1 | 868357 | 20732120 | 23.88 | 3.87 | 34.11 | 8.81 |
| Gbp2 | 867185 | 5713468 | 6.59 | 0.38 | 2.51 | 6.61 |
| Mki67 | 846723 | 1722916 | 2.03 | 0.95 | 2.09 | 2.20 |
| Hmmr | 796488 | 2638066 | 3.31 | 2.46 | 8.66 | 3.52 |
| Spc24 | 726270 | 1658242 | 2.28 | 4.90 | 12.34 | 2.52 |
| Ltbp2 | 687049 | 5748239 | 8.37 | 0.85 | 11.37 | 13.40 |
| Zwint | 639869 | 4229712 | 6.61 | 30.27 | 156.87 | 5.18 |
| Nek6 | 585576 | 2256154 | 3.85 | 4.72 | 12.84 | 2.72 |
| Parvb | 440050 | 1648774 | 3.75 | 0.76 | 1.67 | 2.20 |
| Coq5 | 412104 | 1030297 | 2.50 | 6.11 | 14.49 | 2.37 |
| Grem1 | 397151 | 3486111 | 8.78 | 1.85 | 6.35 | 3.44 |
| Tpx2 | 341218 | 2428677 | 7.12 | 5.50 | 14.22 | 2.59 |
| Fhit | 336059 | 736413 | 2.19 | 3.34 | 7.35 | 2.20 |
| Ube2c | 327086 | 804840 | 2.46 | 29.70 | 82.83 | 2.79 |
| Sncg | 282876 | 1942897 | 6.87 | 1.12 | 13.61 | 12.11 |
| Rab3b | 275670 | 989514 | 3.59 | 0.58 | 1.58 | 2.75 |
| Kif20a | 229068 | 1139925 | 4.98 | 1.83 | 10.05 | 5.51 |
| Wdfy1 | 224811 | 10656230 | 47.40 | 2.10 | 19.22 | 9.14 |
| Pyroxd2 | 203128 | 1065998 | 5.25 | 1.53 | 4.37 | 2.86 |
| Igfbp2 | 168292 | 1435285 | 8.53 | 5.86 | 17.74 | 3.03 |
| Tmod1 | 139475 | 1196372 | 8.58 | 0.91 | 3.00 | 3.30 |
| Prc1 | 125860 | 636219 | 5.05 | 12.02 | 32.39 | 2.69 |
| Erbb2 | 83480 | 183644 | 2.20 | 1.34 | 3.15 | 2.36 |
| Plau | 65157 | 1603249 | 24.61 | 0.82 | 2.25 | 2.75 |
| Ttyh2 | 25759 | 181938 | 7.06 | 7.69 | 15.72 | 2.04 |
| Msr1 | 24735 | 60913 | 2.46 | 6.72 | 17.02 | 2.53 |

**Table. S6**

| No. | Gene Symbol | Ensembl ID | Predicted target* of miR-23-3p | miR-27-3p | miR-24-3p | miR-140-5p |
|---|---|---|---|---|---|---|
| 1 | RAP1B | ENST00000250559.9 | ✓ | ✓ | ✓ | ✓ |
| 2 | NCOA1 | ENST00000405141.1 | ✓ | ✓ | ✓ | ✓ |
| 3 | STRN | ENST00000263918.4 | ✓ | ✓ | ✓ | ✓ |
| 4 | DTNA | ENST00000283365.9 | ✓ | ✓ | ✓ | ✓ |
| 5 | CELF2 | ENST00000379261.4 | ✓ | ✓ | ✓ | ✓ |
| 6 | NLK | ENST00000407008.3 | ✓ | ✓ | ✓ | ✓ |
| 7 | CTCF | ENST00000264010.4 | ✓ | ✓ | | ✓ |
| 8 | YES1 | ENST00000577961.1 | ✓ | ✓ | | ✓ |
| 9 | RAP2B | ENST00000323534.2 | ✓ | ✓ | | ✓ |
| 10 | ABCA1 | ENST00000374736.3 | ✓ | ✓ | | ✓ |
| 11 | PPP1R12A | ENST00000261207.5 | ✓ | ✓ | | ✓ |
| 12 | PAX9 | ENST00000361487.6 | ✓ | ✓ | | ✓ |
| 13 | WDR37 | ENST00000358220.1 | ✓ | ✓ | | ✓ |
| 14 | ADAMTS5 | ENST00000284987.5 | ✓ | ✓ | | ✓ |
| 15 | FOXP2 | ENST00000408937.3 | ✓ | ✓ | | ✓ |
| 16 | LPHN2 | ENST00000370715.1 | ✓ | ✓ | | ✓ |
| 17 | PARD6B | ENST00000371610.2 | ✓ | ✓ | | ✓ |
| 18 | BAZ2B | ENST00000392782.1 | ✓ | ✓ | | ✓ |
| 19 | BMP2K | ENST00000335016.5 | ✓ | ✓ | | ✓ |
| 20 | MFHAS1 | ENST00000276282.6 | ✓ | ✓ | | ✓ |
| 21 | ZHX1 | ENST00000395571.3 | ✓ | ✓ | | ✓ |
| 22 | SATB2 | ENST00000417098.1 | ✓ | ✓ | | ✓ |
| 23 | ANKFY1 | ENST00000341657.4 | ✓ | ✓ | | ✓ |
| 24 | JHDM1D | ENST00000397560.2 | ✓ | ✓ | | ✓ |
| 25 | CELF1 | ENST00000395290.2 | ✓ | ✓ | | ✓ |
| 26 | TEAD1 | ENST00000361905.4 | ✓ | ✓ | | ✓ |
| 27 | PRR14L | ENST00000434485.1 | ✓ | ✓ | | ✓ |
| 28 | WEE1 | ENST00000299613.6 | ✓ | ✓ | | ✓ |
| 29 | STOX2 | ENST00000308497.4 | ✓ | ✓ | | ✓ |
| 30 | MARK1 | ENST00000366918.4 | ✓ | ✓ | | ✓ |
| 31 | TRIM44 | ENST00000299413.5 | ✓ | ✓ | | ✓ |
| 32 | APPBP2 | ENST00000083182.3 | ✓ | ✓ | | ✓ |
| 33 | VCPIP1 | ENST00000310421.4 | ✓ | | ✓ | ✓ |
| 34 | NDST1 | ENST00000261797.6 | ✓ | | ✓ | ✓ |
| 35 | B3GNT1 | ENST00000311181.4 | ✓ | | ✓ | ✓ |
| 36 | YOD1 | ENST00000315927.4 | ✓ | | ✓ | ✓ |
| 37 | SNX27 | ENST00000368843.3 | ✓ | | ✓ | ✓ |
| 38 | SYS1 | ENST00000243918.5 | ✓ | | ✓ | ✓ |
| 39 | LHFPL2 | ENST00000515007.2 | ✓ | | ✓ | ✓ |
| 40 | FAM175B | ENST00000298492.5 | ✓ | | ✓ | ✓ |
| 41 | PURB | ENST00000395699.2 | ✓ | | ✓ | ✓ |
| 42 | FAM46A | ENST00000369754.3 | ✓ | | ✓ | ✓ |
| 43 | ANKRD52 | ENST00000267116.7 | ✓ | | ✓ | ✓ |
| 44 | CCDC85C | ENST00000380243.4 | | ✓ | ✓ | ✓ |
| 45 | HEG1 | ENST00000311127.4 | | ✓ | ✓ | ✓ |
| 46 | TSC22D2 | ENST00000361875.3 | | ✓ | ✓ | ✓ |
| 47 | PDGFRA | ENST00000257290.5 | | ✓ | ✓ | ✓ |
| 48 | ROR1 | ENST00000371079.1 | | ✓ | ✓ | ✓ |
| 49 | ZBTB20 | ENST00000462705.1 | | ✓ | ✓ | ✓ |
| 50 | MED13 | ENST00000397786.2 | | ✓ | ✓ | ✓ |
| 51 | RAD54L2 | ENST00000409535.2 | | ✓ | ✓ | ✓ |
| 52 | RC3H1 | ENST00000367696.2 | | ✓ | ✓ | ✓ |
| 53 | ERC2 | ENST00000288221.6 | | ✓ | ✓ | ✓ |
| 54 | NFAT5 | ENST00000354436.2 | | ✓ | ✓ | ✓ |
| 55 | AP2B1 | ENST00000262325.7 | | ✓ | ✓ | ✓ |

*Source: TargetScan 8.0 https://www.targetscan.org/vert_80/
